# Transcriptional changes associated with breast cancer occur as normal human mammary epithelial cells overcome senescence barriers and become immortalized

**DOI:** 10.1186/1476-4598-6-7

**Published:** 2007-01-18

**Authors:** Yizheng Li, Jing Pan, Jian-Liang Li, Jee Hyung Lee, Chris Tunkey, Katie Saraf, James C Garbe, Maryann Z Whitley, Scott A Jelinsky, Martha R Stampfer, Steven A Haney

**Affiliations:** 1 Section of Bioinformatics, Department of Biological Technologies, Wyeth Research, 87 Cambridge Park Drive, Cambridge, MA 02140, USA; 2 Applied Genomics, Department of Biological Technologies, Wyeth Research, 87 Cambridge Park Drive, Cambridge, MA 02140, USA; 3 Molecular Profiling and Biomarker Discovery, Department of Biological Technologies, Wyeth Research, 87 Cambridge Park Drive, Cambridge, MA 02140, USA; 4 Life Sciences Division, Lawrence Berkeley National Laboratory, Berkeley, CA 94720, USA

## Abstract

**Background:**

Human mammary epithelial cells (HMEC) overcome two well-characterized genetic and epigenetic barriers as they progress from primary cells to fully immortalized cell lines *in vitro*. Finite lifespan HMEC overcome an Rb-mediated stress-associated senescence barrier (stasis), and a stringent, telomere-length dependent, barrier (agonescence or crisis, depending on p53 status). HMEC that have overcome the second senescence barrier are immortalized.

**Methods:**

We have characterized pre-stasis, post-selection (post-stasis, with p16 silenced), and fully immortalized HMEC by transcription profiling and RT-PCR. Four pre-stasis and seven post-selection HMEC samples, along with 10 representatives of fully immortalized breast epithelial cell lines, were profiled using Affymetrix U133A/B chips and compared using both supervised and unsupervised clustering. Datasets were validated by RT-PCR for a select set of genes. Quantitative immunofluorescence was used to assess changes in transcriptional regulators associated with the gene expression changes.

**Results:**

The most dramatic and uniform changes we observed were in a set of about 30 genes that are characterized as a "cancer proliferation cluster," which includes genes expressed during mitosis (*CDC2*, *CDC25*, *MCM2*, *PLK1*) and following DNA damage. The increased expression of these genes was particularly concordant in the fully immortalized lines. Additional changes were observed in IFN-regulated genes in some post-selection and fully immortalized cultures. Nuclear localization was observed for several transcriptional regulators associated with expression of these genes in post-selection and immortalized HMEC, including Rb, Myc, BRCA1, HDAC3 and SP1.

**Conclusion:**

Gene expression profiles and cytological changes in related transcriptional regulators indicate that immortalized HMEC resemble non-invasive breast cancers, such as ductal and lobular carcinomas *in situ*, and are strikingly distinct from finite-lifespan HMEC, particularly with regard to genes involved in proliferation, cell cycle regulation, chromosome structure and the DNA damage response. The comparison of HMEC profiles with lines harboring oncogenic changes (e.g. overexpression of Her-2^neu^, loss of p53 expression) identifies genes involved in tissue remodeling as well as proinflamatory cytokines and S100 proteins. Studies on carcinogenesis using immortalized cell lines as starting points or "normal" controls need to account for the significant pre-existing genetic and epigenetic changes inherent in such lines before results can be broadly interpreted.

## Background

Genetic and epigenetic changes that occur early in the process of carcinogenesis may enable the survival and growth of cells that subsequently acquire oncogenic mutations. One early alteration in the development of human carcinomas is the acquisition of an immortal potential, associated with reactivation of endogenous *hTERT *expression and maintenance of stable telomere lengths. [1]. We have employed an *in vitro *HMEC model system to examine gene expression changes during the process of transformation of normal finite cells to immortality and malignancy [2-11]. Two mechanistically distinct barriers to unlimited proliferation have been described. The first barrier, stasis (stress-associated senescence) is associated with elevated levels of the cyclin-dependent kinase inhibitor (CKI) p16^INK4A ^[6]. Stasis appears to be Rb-mediated and not directly dependent on telomere length. Cells arrested at this barrier exhibit a viable G1 arrest with a low labeling index (LI), normal karyotypes, expression of senescence -associated ß-galactosidase (SA-ß-gal) activity, and a senescent morphology [7,12]. HMEC can undergo a variable number of population doublings (PD), depending upon culture conditions, prior to encountering stasis.

Multiple types of single changes that prevent Rb-mediated growth inhibition will overcome stasis. Loss of *CDKN2A *(encoding p16^ink4a^) expression, from methylation-induced *CDKN2A *promoter silencing, or mutations, is one alteration frequently observed in human breast cancers and cultured HMEC [6,13,14]. HMEC cultured in a serum-free medium can produce rare cells that spontaneously silence the p16 promoter and resume growth, a process termed selection, with the resulting post-stasis population called post-selection [3]. In the HMEC, no increase in p53, p21, or p14^ARF ^levels have been seen at stasis [7] and p53 function is not required for the stasis barrier (J.G. and M.S., unpublished). Rare HMEC with silenced p16 are also observed in vivo and have been called variant HMEC (vHMEC) [15,16].

HMEC that have overcome or bypassed stasis encounter a second barrier as a consequence of telomere dysfunction. Ongoing proliferation in the absence of telomerase expression leads to critically shortened telomeres, and chromosomal aberrations [7,17]. In post-selection HMEC with functional p53, these aberrations induce a mostly viable G1 and G2 arrest (termed agonescence); if p53 is non-functional, massive cell death (crisis) ensues (J.G. and M.S., unpublished) [18]. Telomere dysfunction poses an extremely stringent barrier to human cellular immortalization; in post-selection HMEC multiple errors appear to be necessary for telomerase reactivation, and immortalization [4,8]. Since this barrier is dependent upon telomere length, ectopic overexpression of hTERT readily immortalizes post-selection HMEC [19]. HMEC can be immortalized using several different pathologically relevant agents, e.g., chemical carcinogens, over-expression of the breast cancer-associated oncogenes *c*-*myc *and/or *ZNF217*, and/or inactivation of p53 function [8,9,11]. Fully immortal HMEC maintain telomeres at short, stable lengths, but do not necessarily express malignancy-associated properties; overexpression of specific oncogenes can confer malignant properties [20-22].

Transcriptional profiling has proven to be a valuable technology for describing the differences between cell types and experimental treatments for many disease models, particularly cancer [23]. One of the most well-developed stratifications of human cancers has been for breast cancer [24,25]. These and other studies have shown that a common set of genes is consistently overexpressed in most cancers [26], including many cell cycle regulated genes and genes required for mitosis (e.g. *MKI67*, *PCNA*, *BIRC5*, *MYBL2*, *TOP2A*, *PLK1*, *MCM2*-*MCM6*, *CDC20*). The frequent identification of these genes in cancer cells suggests that they represent a common characteristic of cancers, irrespective of the cell type from which the cancers originate.

The data described here examines the changes that occur as HMEC overcome the barriers to indefinite proliferation. We show that pre-stasis and post-selection HMEC are profoundly different from fully immortalized HMEC lines, despite the fact that the immortalized lines may retain normal growth factor requirements, lack anchorage-independent growth or invasiveness, and are not tumorigenic in animal models [4]. Rather, the non-malignant immortalized lines display the cancer-associated proliferation cluster of genes frequently identified in transcriptional profiling studies of cancer cells and tissues [26].

## Materials and methods

### Reagents and supplies

MEBM serum-free medium was purchased from the Clonetics division of Cambrex BioScience (Walkersville, MD), and was supplemented with EGF, hydrocortisone, insulin, and BPE using Singlequot reagent packs from Clonetics, as well as 5 μg/ml transferrin (Clonetics) and 10 nM isopeterenol (Sigma). Hams F-12/DMEM (50:50) was purchased from Invitrogen or prepared by Core Technical Services (Wyeth Research), and supplemented to contain 5% FBS (Invitrogen), 2 mM pyruvate (Invitrogen), 2 mM glutamine (Invitrogen), 20 ng/ml EGF (Clonetics), 200 μg/ml cholera toxin (Sigma), 1× ITS (Clonetics), 500 ng/ml hydrocortisone (Sigma or Clonetics), and 20 mg/ml gentamycin (Invitrogen). MM medium was prepared as described [2]. Antibodies and fluorescent dyes used in High Content Screening (HCS, or quantitiative immunofluorescence) were obtained from Cell Signaling Technologies (Beverly, MA), Upstate Biotechnologies (Lake Placid, NY), and Molecular Probes/Invitrogen (Carlsbad, CA), as described in the supplementary material. Antibodies were screened by Western blot prior to immunofluorescence studies to verify that they recognize a single specific antigen of the expected molecular size.

### Cell culture

Pre-stasis and post-selection HMEC, from specimens 48, 161, 184, 191, 195 and 239, as well as the immortally transformed lines 184A1, 184AA2, 184AA3, 184B5 were developed and characterized at LBNL, starting with reduction mammoplasty tissues; an additional post-selection HMEC strain was obtained from Clonetics. Remaining lines, as well as additional samples of 184A1 and 184B5 were obtained from ATCC (Manassas, VA). 184B5ME was derived from immortal 184B5 following stable expression of *ERBB2/Her2 *and selection for anchorage independent growth (Stampfer, unpublished). Pre-stasis cells were maintained in MM media [2], and post-selection cells were maintained in MEBM prior to this study. Pre-stasis HMEC display 15–25 PD in MM, and 10–15 PD in MEBM, prior to growth arrest at stasis. For transcriptional profiling studies, all lines maintained at LBNL (listed above), as well as the post-selection HMEC purchased from Clonetics, were revived in MEBM media and cultured at 37°C with 1% CO_2_. Consequently, the pre-stasis HMEC were studied as they neared stasis. Pre-stasis HMEC used in HCS were cultured in MM medium. Fully immortalized cell lines obtained from ATCC (184A1, 184B5, MCF10A, MCF10A-2 and MCF12A) were cultured in DMEM/Ham's F-12 medium, at 37°C with 10% CO_2_, as they were maintained prior to crypreservation.

### RNA labeling, GeneChip hybridizations and expression analysis

Cells to be prepared for RNA extraction were revived from cryopreservation and cultured to 80% confluence in a single T-75 flask, trypsinized under conditions appropriate for each line, and split 1:4 into four new T-75 flasks. When cells reached 80% confluence three of the flasks were trypsinized, lysed and total RNA isolated using the Midiprep RNA isolation kit from Qiagen, according to manufacturers instructions.

An 11-point standard curve of bacterial cRNA control samples was added prior to hybridization as described [27,28]. Three independent replicates were generated per cell type at the indicated stage. Affymetrix's MAS5 algorithm was used to generate expression measures including Signal values and Absent/Present calls (Affymetrix (2001) *Microarray Suite User Guide*, Version 5. [29]. A global scaling normalization was applied to the raw signal intensity. Briefly, a 2% trimmed-mean was calculated per chip, and was scaled to an arbitrary value of 100. A scaled Signal value was then computed for each gene by multiplying its original Signal intensity with the scale factor (100/trimmed-mean). Subsequently, genes were filtered to remove those with uninformative or noisy expression changes across the entire samples. A gene is selected for downstream analysis if its expression exceeds 50 (scaled) Signal unit in at least one sample. Analysis of variance (ANOVA) was performed with log2 transformation on the scaled Signals of several cell lineage groups (see details below). Data was analyzed using several analytical approaches, including unsupervised clustering [30], supervised clustering [31,32], and principal components analysis. For the unsupervised clustering, genes that are filtered based on the Pvalues from one-way analysis of variance (ANOVA) on four cell lineage groups as well as greater than 2 fold difference among the four groups. These groups consist of 1) all finite lifespan cells, 2) *p53*^+/+ ^immortalized 184A1 and 184B5, 3) *p53*^-/- ^immortalized 184AA2 and 184AA3, and 4) immortalized non-184 derived cells (including MCF10A, MCF10A-2, and MCF12A).

### Promoter analysis

Genes identified as unique classes in a subset of post-selection HMEC were examined in detail (see Results for a complete list of genes). Initially, the 500 bp upstream of the transcription start site for each gene was examined for well-characterized transcription binding sites using two algorithms, Match and Clover [33,34]. For most of the groups, strong assignments of specific promoter binding sites could be identified using both algorithms. One class (Class B in the Results) was less definitive, so the region was extended to 2 kb prior to the transcription start site for those genes.

### Taqman™ quantitative PCR

Primer sets for 15 genes analyzed by Taqman™ analysis were obtained from Applied Biosystems (Foster City, CA) and used according to standard protocols. Genes tested are listed in the Results section.

### High content screening

Cells were seeded at 5000 cells/well in a 96-well black wall, clear bottom Packard ViewPlate, and incubated in MM, MEBM or DMEM/F-12 medium for pre-stasis, post-selection and immortalized HMEC, respectively, for 48 hours. Cells were washed with PBS, and fixed with pre-warmed 4% paraformaldehyde for 10 minutes. Cells were washed 2× with PBS, permeabilized with 0.2% Triton X-100 for 3–5 minutes, and washed 2× with PBS again. Cells were stained with primary antibodies in 1% BSA/PBS. Primary antibodies were used as follows: E2F1 (BD/Pharmagin, 1:200 dilution), E2F4 (Abcam, 1:400), Rb (Cell Signaling Technologies, 1:400), p107 (Santa Cruz, 1:200), BRCA1 (Abcam, 1:200), p53 (Cell Signaling Technologies, 1:200), SP1 (Upstate Biotechnologies, 1:400), NF-κB (Cellomics, 1:200). Cells were washed 3× with PBST (0.05% Tween-20), and stained with DAPI and secondary antibodies of appropriate species/isotype specificity and conjugated to either Alexa-488 or Alexa-594. Cells were washed again 3× with PBST; 100 μl of PBS was added and plates were sealed with an adhesive cover.

Quantitative immunofluorescence was performed using a Cellomics ArrayScan V^ti^. Images were taken using a 20× objective and data was collected for a minimum of 1000 valid cells per well. Valid cells are defined as having nuclei with expected DNA content (defined by DAPI fluorescence intensity), nuclei size and shape typical for the cell line/type, and well-separated from neighboring cells, such that cytoplasmic regions could be clearly resolved. DNA content and antigen intensity were quantitated for each cell, and the nuclear-cytoplasmic ratio for each antigen was determined by a mask derived from the DAPI staining, which was used to define the nucleus, and a region surrounding the nucleus (which was specific for each cell line/type) was used to define the cytoplasm. Quantitation was performed using either the Compartmental Analysis or Nuclear Translocation BioApplications, from Cellomics.

## Results

### Transcriptional profiling of pre-stasis, post-selection and immortalized HMEC

**Figure 1:**
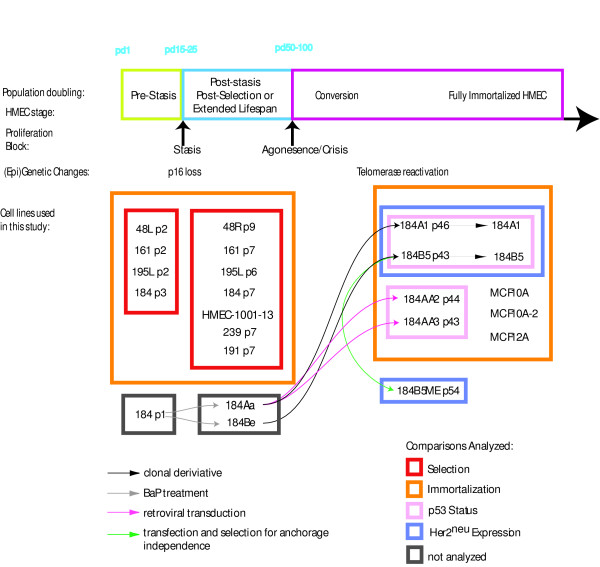
***Graphic relationship of cell lines profiled in this study***. Cell lines characterized in this study are shown with reference to their stage in transformation. The pre-stasis HMEC used were cultured for 2–3 passages before analysis, and reach stasis by passages 3–5. Rare isolates of cells grown in serum-free media (MEBM) emerge spontaneously from stasis, associated with the absence of p16 expression due to promoter silencing, and continue growing as post-selection HMEC until reaching a second, proliferation barrier (telomere dysfunction). This barrier is highly stringent, and spontaneous immortalization has never been observed in cells that were not mutagenized or virally transduced during pre-stasis or post-selection growth. HMEC grown in MM do not spontaneously give rise to post-selection cells, however primary populations exposed to the chemical carcinogen benzo(a)pyrene (BaP) have produced rare clonal isolates with post-stasis growth, associated with absence of p16 expression due to mutation or promoter silencing. These non-spontaneously arising post-stasis cells are referred to as *extended lifespan*, and may harbor additional errors due to the carcinogen exposure. Overcoming the telomere dysfunction barrier is associated with reactivation of telomerase activity. The fully immortalized lines 184A1 and 184B5 were derived from extended lifespan post-stasis cells grown in MM and exposed to BaP in primary culture. Exposure of extended lifespan 184Aa cells to retroviral infection resulted in two cell lines that had lost both copies of the *TP53 *gene. The cell lines profiled in this study are shown relative to the profiling analyses performed. Comparisons used to analyze selection and immortalization, as well as the influence of p53 and ERBB2/Her2 status are shown by colored boxes and identified in the key at the lower left of the figure.

To better understand the extent to which pre-stasis, post-selection and immortalized HMEC represent distinct cell types, we compared several samples of these cultures by transcriptional profiling; the HMEC samples characterized are described in Table 1. The finite lifespan pre-stasis and post-selection HMEC are referred to as strains or cell types from a specific source, and culture conditions (including stage) are noted for each particular sample. The relationships between samples in this study, their origins, are indicated graphically in Figure 1. Triplicate cultures for each sample were grown under the conditions indicated in the Methods, and in Table 1, following which the total RNA was isolated, labeled and hybridized to the Affymetrix U133A/B GeneChips.

**Table 1:** Cell Types and Lines Used in This Study

**Cell Name**	**Source**	**Stage**	**Growth Media**
48L	LBNL	Pre-stasis, finite lifespan strain	MM (MEBM)***
161	LBNL	Pre-stasis, finite lifespan strain	MM (MEBM)
184	LBNL	Pre-stasis, finite lifespan strain	MM (MEBM)
195L	LBNL	Pre-stasis, finite lifespan strain	MM (MEBM)
48R	LBNL	Post-selection, finite lifespan strain	MEBM
161	LBNL	Post-selection, finite lifespan strain	MEBM
184	LBNL	Post-selection, finite lifespan strain	MEBM
195L	LBNL	Post-selection, finite lifespan strain	MEBM
191	LBNL	Post-selection, finite lifespan strain	MEBM
239	LBNL	Post-selection, finite lifespan strain	MEBM
HMEC-1001-13	Clonetics	Post-selection, finite lifespan strain**	MEBM
184A1	LBNL	Fully immortal cell line	MEBM
184B5	LBNL	Fully immortal cell line	MEBM
184AA2	LBNL	Fully immortal cell line	MEBM
184AA3	LBNL	Fully immortal cell line	MEBM
184B5ME	LBNL	Fully immortal cell line	MEBM
184A1*	ATCC	Fully immortal cell line	DMEM/F-12
184B5*	ATCC	Fully immortal cell line	DMEM/F-12
MCF-10A	ATCC	Fully immortal cell line	DMEM/F-12
MCF-10A-2	ATCC	Fully immortal cell line	DMEM/F-12
MCF-12A	ATCC	Fully immortal cell line	DMEM/F-12

*designated as 184A1(a) and 184B5(a) in other tables and figures

**stage defined by transcriptional profile

***cells isolated from reduction mammoplasty tissues and expanded in MM media to passages 2–3, then cultured in serum-free MEBM media for transcriptional profiling

**Figure 2:**
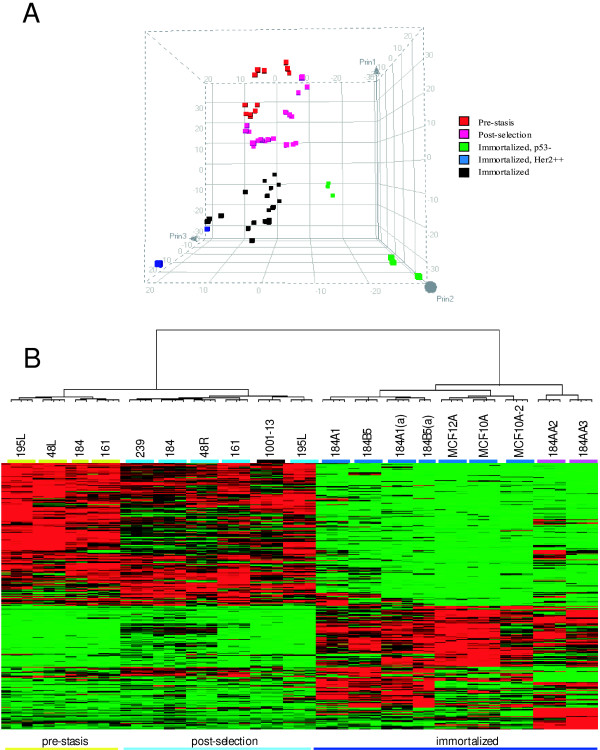
***Relationship of HMEC as determined by transcriptional profiles***. **A**. Data from 2319 genes were used to determine the number of principal components of the data. Three components were identified, and the contribution of the components to the transcription profile of each cell line samples are shown in the figure. Individual replicates for each cell line are shown. Cell lines grouped in **Figure 1 **are shown in **Figure 2A **as shown in the legend. Vertical axis is PC1, the first, and therefore the strongest. principal component. **B**. Unsupervised clustering of HMEC. All genes that change expression in one or more samples were used to cluster the cell types and lines by overall similarity. Cell types and lines are identified by color under the designations: pre-stasis HMEC: light green; post-selection HMEC: light blue; fully immortalized HMEC: dark blue; p53^-/- ^fully immortal HMEC: burgundy, and lines not formally characterized: black. Samples of 184A1 and 184B5 designated by (a) were obtained from ATCC.

Principal Component Analysis (PCA) was used to visualize the gross relationships among the cell types, as shown in Figure 2A. The first three components, which explains about 60% of the total variation, are displayed in a three dimensional graph. The pre-stasis HMEC (in red) and post-selection HMEC (in pink) are clearly separated from the immortalized lines (in blue, black and green) along the first principal component axis. Thus, transcriptional profiling defines the transition from finite lifespan to fully immortalized HMEC as the most significant change in HMEC progression. The pre-stasis and post-selection HMEC are also well segregated within their unique space. In addition, the fully immortalized lines that either do not express *p53 *or are transduced with *ERBB2/Her2 *(green and blue, respectively) are distinguished from the rest of the immortalized lines (black). According to the PCA, there are no significant differences between the fully immortalized lines derived from various methods of immortalization, or from lines maintained at LBNL versus those obtained from ATCC. Unsupervised (or Eisen) clustering of the genes that change following selection and immortalization for most of the samples is shown in Figure 2B. These data reflect the 1 342 genes that are filtered based on the Pvalues from one-way analysis of variance (ANOVA), as described in the supplementary material.

### Gene expression changes following selection

**Figure 3:**
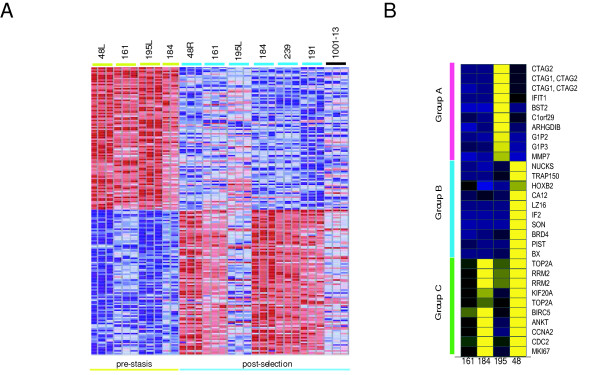
***Supervised Clustering of Pre-stasis, and Post-selection HMEC***. **A**. Gene expression values were normalized and characterized for the significance of overexpression in one group relative to other groups in the comparison. The top 50 genes that are significantly overexpressed in one group are shown. All pre-stasis and post-selection cell types have been used. Analysis was performed in GeneCluster, and the color bar describing how normalized values are depicted is shown at the bottom of the figure. **B**. Distinct classes of genes over-expressed in post-selection HMEC. Genes showing one of three specific patterns of expression in the four pairs of pre-stasis and post-selection samples are diagramed. The top 10 qualifiers (based on fold change) are shown (some genes are represented by more than one qualifier).

Gene expression changes that distinguish pre-stasis from post-selection cells were identified using GeneCluster [31], and the results are shown in Figure 3A. The figure characterizes a large set of concordantly-regulated genes in the pre-stasis strains, and a high level of concordance in four of the six post-selection HMEC (200 genes for each class). Among these top-200 genes in the pre-stasis cell types, the largest number of genes we identified are involved in the extra-cellular matrix (ECM), including structural proteins and matrix remodeling enzymes (listed in supplementary Additional file 1). Examples include many collagen and kallikrein genes. Genes that increase expression level in post-selection HMEC include a large number of genes associated with proliferation and the cell cycle. These genes are strongly associated with cancer cell growth, and increase in expression directly with tumor grade. Specific examples include *BIRC5*, A and B type cyclin genes, *CDC2*, and the *MCM *chromosomal proteins. The increased expression of these genes is dependent on E2F transcription factors and reflects the proliferative state a cell. Since the pre-stasis cells were nearing stasis, the increased expression of the genes in the post-selection HMEC may reflect either a loss of Rb repression (consistent with a loss of p16), or could reflect the relative proliferative state of these pre-stasis and post-selection cells.

The two discordant post-selection HMEC we observed in Figure 3A (195L and 1001-13), suggest that additional molecular events can occur during selection; these samples also show a loss of p16 expression (results not shown), a definitive event for post-selection HMEC. In order to probe further into the changes that occur during selection, we compared the four sets of HMEC studied as pre-stasis and post-selection samples. For this analysis, we identified genes that increase expression in post-selection HMEC, as compared to the corresponding pre-selection sample. Four patterns were observed. The genes we identified in each group are listed in Table 2, and the expression changes we observe for three of the groups are shown in Figure 3B. The group not explicitly shown in Figure 3B is uniformly down-regulated in all four pairs. Genes expressed exclusively in post-selection 195L HMEC (Group A) fall into two categories: genes previously identified as cancer-associated (including several antigens proposed as cancer biomarkers), and genes induced by interferons [35]. Among the cancer-associated genes, the *Cancer-Testis Antigen 2 *(*CTAG-2*) is very strongly expressed (30-fold according to the GeneChip data), as are *ARHGDIB*/*Ly-GDI*, and *IGFBP6*. The cytokine induced genes [35] include a set previously reported as increasing in post-selection HMEC, such as *IFIT1*, *IFITM1*, *G1P2 *and *OAS1 *[36]. The genes that are unique to 48 HMEC (Group B) include several transcription factors and cell cycle proteins whose roles in cancer or breast tissue development have not been well characterized to date, including *NUCKS*, *SON *and *HOXB2*. Group C includes many genes previously associated with cancer cell proliferation.

**Table 2:** Genes and Promoter Elements That Define Post-Selection HMEC Gene Expression Classes

Geneset Classes	Genes	Promoter Elements
Group A		
IFN genes	IFIT1, BST2, G1P2, G1P3, IFIT2, OAS1, IFI44, IFIT4	IRF, ILR, IRL
Non-IFN genes	CTAG2, ARHGDI-B/Ly-GDI, MMP7, PLAU, CALB1, SLC1A6, MDA5, FXYD5, HMOX1	
Group B	NUCKS, HDAC3, TRAP150, HOXB2, SON, IF2, LZ16, ANLN, BBX, TOP1, H4FG, SFRP1, KTN1, GTAR, BAZ1A, PK428, FALZ, TTC3, DNCL12, RBM9	SP1
Group C	TOP2A, RRM2, KIF20A, BIRC5, ANKT, CCNA2, CDC2, MKI67, CDC20, MCM5, HMMR, IL-1B, PRC1, PMSCL1, MADL1, DLG7	E2F, NF-Y, B-Myb
Group D	H11, COL11A1, IGFBP5, CNN1, COMP, LGALS7, CLDN7, KLK6, KLK7, KLK10, KLK11 KRT23, LOXL4, THY1, FLJ21841	MAZ, MAZR, MEF-3

Since these geneset classes were comprised of a relatively small number of genes, we performed promoter analyses, to see if these sets are linked in specific pathways. Promoter binding sites we were able to identify are listed in Table 2. For Group A, interferon-responsive elements were found for most of the genes, but not the cancer/metastasis-associated genes (*BST2 *is an exception), consistent with previous studies that did not identify these genes as IFN-regulated [35]. Instead, several genes in this group have been shown to be direct or indirect targets of p53 and Myc. A common element in the regulation of both p53/Myc and IFN-regulated genes is *BRCA1*, and in particular, BRCA1 is essential for the activation of stress and inflammatory response genes following treatment with interferons [37]. Group B was less well-defined by specific binding sites near the promoter, but an extended analysis (2 kb) identified SP1, E2F, MAZ and NF-Y binding sites for many genes. These binding sites were also identified in the genes of Group C, especially the E2F, NF-Y and SP1 sites, which is consistent previous work [38,39]. Group D, genes significantly repressed in post-selection HMEC, may be under the control of MAZ (Myc-associated zinc finger protein), as binding sites were found in 19 of 22 genes examined, which is consistent with previous observations that increased Myc can repress ECM genes [40-42]. In conclusion, although distinct gene expression patterns could be observed for each of the pre-stasis/post-selection HMEC pairs we have characterized, in each case strong associations could be made between the promoters of each class and the proliferation and cell cycle transcription factors, particularly E2F, SP-1, NF-Y and the Myc-related MAZ. The distinguishing features for each of these expression classes is likely to be found in additional, unique pathways such as BRCA1-mediated regulation.

### Gene expression changes that distinguish finite life span HMEC from immortally transformed HMEC

**Figure 4:**
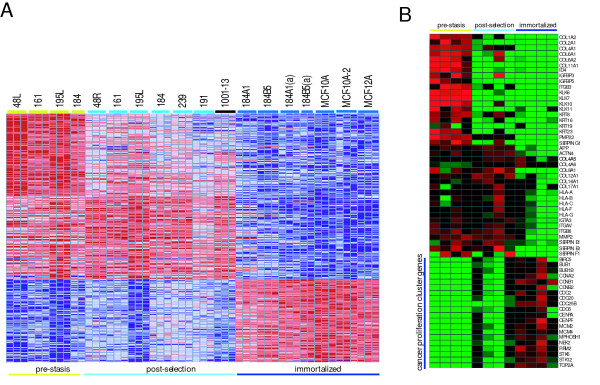
***Supervised Clustering of Pre-stasis, Post-selection and Immortalized HMEC***. **A**. Gene expression values were normalized and characterized for the significance of over-expression in one group relative to other groups in the comparison. The top 200 genes (of 1342) that are significantly over-expressed in one group are shown. All pre-stasis, post-selection and immortalized HMEC (except the *p53*^-/- ^and *ERBB2/Her2 *transfected variants) have been grouped. The top 100 genes (of 1440) that are over-expressed in one group relative to the other two are presented. Analysis was performed in GeneCluster. **B**. *Expression of a subset of highly concordant genes in pre-stasis, post-selection and fully immortalized HMEC*. Gene-normalized expression of 60 genes identified in the figure are shown for four representatives each for the three groups of HMEC. Samples are (left to right): 48L, 161, 195L and 184; 48R, 161, 195L, and 184; 184A1, 184B5, MCF-10A and MCF12A.

The most significant transition observed in this study is that of immortalization. Genes whose expression are reduced in the immortalized lines include a significant number that suppress angiogenesis, contribute to the ECM, or regulate the actin cytoskeleton. Many of these genes were identified as down-regulated in HMEC following selection as well; some are further down-regulated in the immortalized lines, as shown in Figure 4A. These comparisons include multiple independent samples from each stage, including four distinct fully immortalized cell lines, and three additional samples from either different sources (184A1 and 184B5 from ATCC) or two separate isolates from the same experiment (MCF-10A and MCF-10A-2) [43]. The genes identified in each group are described in Additional file 2. Collectively, the pre-stasis and post-selection samples are distinguished most strongly by changes to the ECM and cell-cell communication genes, particularly collagens, kallikrein, matrix metalloproteinase and serpin proteinases; genes that affect the actin cytoskeleton are also noted (both actin and actin-interactors, such as actinin, nidogen, transgelin, and palladin, genes). Several well-recognized classes of genes are up-regulated in fully immortalized lines, including the commonly observed "proliferation cluster" described above. These genes were also observed to be up-regulated in the post-selection, compared to pre-stasis HMEC. Fewer of these "proliferation genes" are identified in the fully immortalized samples following a three-way comparison, but this is because GeneCluster identifies the most definitive group of genes for each class, and since some of the post-selection samples express increased levels of genes such as *MCM2 *and *STK12*, they are not unique to either the post-selection or the fully immortalized HMEC.

We have examined the expression of the cancer cell proliferation class of genes directly in Figure 4B. In this example, the absolute expression levels of each gene listed in the figure are displayed directly (rather than the ratio of post-selection over pre-stasis expression levels in Figure 3B). These genes are compared to equal subsets of genes that show maximal levels of expression in the pre-stasis and post-selection HMEC samples. As can be observed in the figure, genes showing maximal expression in the pre-stasis samples are robust, whereas those showing maximal expression in the post-selection are less strongly definitive of post-selection cells. The "proliferation cluster" genes show strongest expression in the fully immortalized HMEC lines, however expression of these genes is heterogeneous for both the post-selection and fully immortalized sets. Increased expression can be observed for the post-selection 48R and 184 samples (as was seen for some of these genes in Figure 3B), and lesser expression is seen for MCF-12A. However, the rise in expression of this group of genes as HMEC progress from pre-stasis through fully immortalized stages is clear.

### Gene expression changes observed in p53^-/-^ cell lines

HMEC lines that have lost p53 during immortalization show distinctive changes in transcriptional profiles when compared to closely related lines that have retained p53 function. The complete list of genes is presented in the supplementary Additional file 3. When we explicitly look for genes whose expression changes are common to the p53 status of the lines derived from specimen 184 cells, several genes showing concordant changes between *p53*^+/+ ^184A1 and 184B5 versus *p53*^-/- ^184AA2 and 184AA3 are observed. *SIAH2*, *Lipocalin 2*, *Asparagine synthase *and *Keratin 15 *are all upregulated in both 184AA2 and 184AA3, relative to both 184A1 and184B5. Genes down-regulated in the p53^-/- ^lines include several that are explicitly regulated by p53 (including *RRM2 *and *TP53INP1*). A comparison of the two *p53*^+/+ ^and the two *p53*^-/- ^lines shows that additional gene expression changes unique to each line have occurred. Examples include *DUSP1 *and *BIRC3*, expressed at significantly higher levels 184AA3 than in 184AA2, and *FABP4*, *IFI27*, *HRASLS3*, and *Fibulin 1*, expressed much more robustly in 184A1 than in 184B5. The complete list of genes is presented in the supplementary Additional file 4 and Additional file 5.

### Gene expression changes resulting from ectopic expression of Her2

The events characterized thus far in this study concern HMEC immortalization; however, additional events are critical to malignancy. To connect these studies directly to changes that occur following an oncogenic event, we have compared one immortalized HMEC line, 184B5, with a derivative that ectopically expresses the *ERBB2*/*Her2 *oncogene, 184B5ME. *ERBB2/Her2 *is frequently over-expressed in breast cancer, and is transforming simply by being over-expressed, so this line models clinically relevant features of breast cancer. Over-expression of *ERBB2/Her2 *in 184B5 results in anchorage independent growth, a malignancy-associated property, while over-expression of oncogenic *ERBB2/Her2 *in 184B5 can confer tumorigenicity [21]. Gene expression changes seen for 184B5ME that are distinct from its parent are listed in the supplementary Additional file 6. Genes showing increased expression include many that were down-regulated in post-selection HMEC, including kallikreins *KLK6 *and *KLK7*, and *cystatin E/M*. These phenotypic reversions may play a role in the transition to invasive cancer [44]. Additional gene expression changes include a dramatic increase in the expression of *IL24 *and significant changes in *BIRC3*, *HRASLS3*, and *PTGES*. Genes showing down-regulation as a consequence of *ERBB2/Her2 *overexpression include many of the IFN genes that showed increased expression following selection (in 195L) or immortalization (in 184A1, 184B5 and others).

### Real-time PCR measurement for selected genes identified in this study

**Figure 5:**
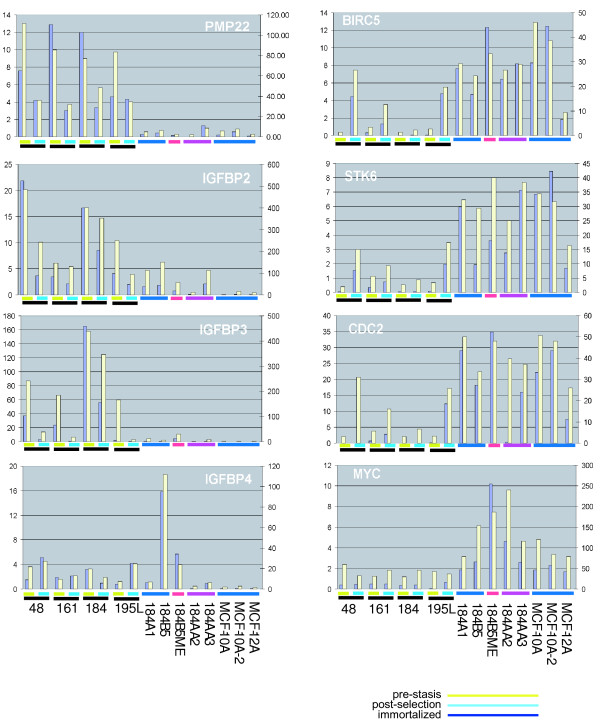
***Real-time PCR measurements of gene identified in transcriptional profiling analyses***. Representative genes from groups identified as changing expression during selection or immortalization were characterized by real-time PCR analysis (TaqMan™). Genes were selected as representative of classes were described in this study. Each gene is presented as a separate graph, as identified in the figure. Cell lines are presented in the same order in each graph, as listed in the bottom left panel. The finite lifespan samples are shown as pairs, with the pre-stasis sample on the left and the post-selection sample on the right. For each cell line, expression data from Affymetrix GeneChips are shown as blue bars, according to the scale at the left of the graphs. Expression data from real-time PCR of the same samples are shown as yellow bars, according to the scale at the right of the graphs.

The results presented comprise a large study of human mammary cell samples that have not been characterized by transcriptional profiling previously, and the gene expression patterns are either new or not previously associated with non-cancerous cell lines. As such we wished to validate the findings by corroborating the gene expression changes observed by genechips with an independent method. 15 genes were chosen from the data to be validated by Taqman™ quantitative PCR. Genes that change following selection (*PMP22/GAS3 *and several insulin-like growth factor binding protein (IGFBP) genes: *IGFBP2*, *IGFBP3*, *IGFBP4*, *IGFBP5*, *IGFBP6*, and *IGFBP7*), as well as genes that change in immortalized lines (*CCNB1*, *CDC2*, *CDC25B*, *HDAC3*, *MYC*, and *STK6*) were evaluated by RT-PCR in 17 cell types, comprising pre-stasis, post-selection and fully immortalized samples, and the results compared to expression data from the oligonucleotide arrays. The concordance between expression of a gene as measured by oligonucleotide array and Taqman™ assays were generally quite good; in 14 cases, only minor discordances can be observed (see Figure 5). *HDAC3 *was an exception. The expression level changes of three probes sets for *HDAC3 *on the Affymetrix U133 GeneArrays, and the Taqman™ primer set, were highly discordant, so we were not able to validate the expression changes of this gene by RT-PCR, however were able to show significant changes in HDAC3 protein expression and localization by immunofluorescence microscopy (described below).

### Transcriptional regulatory factors are localized to the nucleus following selection and immortalization

**Figure 6:**
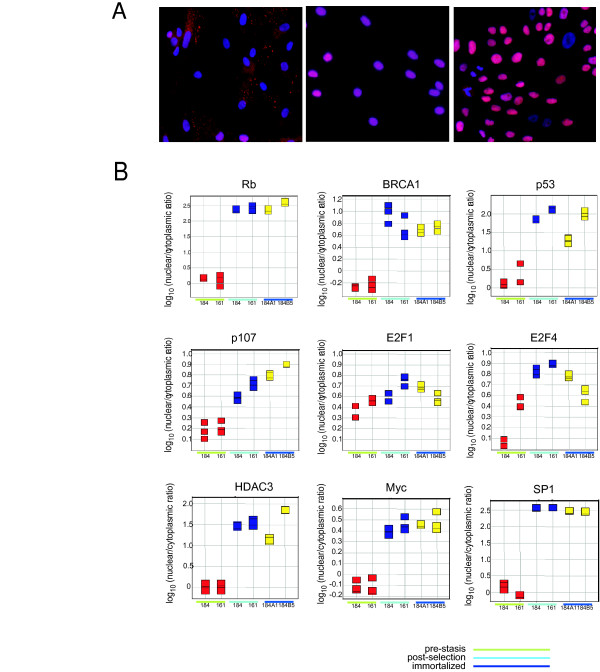
***High Content Screening of proteins associated with cell cycle progression and chromosomal stability***. (A) Immunofluorescent images of Rb (red) and DNA (blue) obtained using a Cellomics ArrayScan Vti are shown for pre-stasis 184 HMEC (left), post-selection 184 HMEC (center) and the 184A1 cell line (right). (B) Quantitation of the nuclear/cytoplasmic ratio is shown for pre-stasis 184 and 161 HMEC, post-stasis 184 and 161 HMEC and the cell lines 184A1 and 184B5, as indicated in the figure panels. Antigens quantitated in each panel are identified above the panel.

We explored the changes that occur in several critical regulators of cell cycle progression and chromosomal stability by quantitative fluorescence microscopy, or High Content Screening (HCS). These factors were chosen based on patterns observed in the transcription profiling data as ones that would be expected to change as HMEC progress past senescence barriers, based on the gene expression patterns we observe. Example images are shown in Figure 6A. For these images, Rb is shown in red and DNA is shown in blue. In the pre-stasis 184 HMEC, Rb is punctate and is evenly distributed between the nucleus and cytoplasm. In post-selection 184 HMEC and in immortalized lines such as 184A1 (shown in the figure) and 184B5 (not shown), Rb is very strongly localized to the nucleus, and the staining is no longer punctate. The nuclear/cytoplasmic ratio (determined using least 1000 cells per sample for three samples each) are shown in Figure 6B for Rb and 8 other proteins. The ratio for Rb in pre-stasis cells is 0.5–2, whereas for post-selection and immortalized HMEC it is greater than 100. Similar dramatic changes are observed for HDAC3, BRCA1, p53 and the general transcription factor SP1. BRCA1 and c-Myc are localized in the cytoplasm in pre-stasis HMEC, but to the nucleus in post-selection and immortalized HMEC. For other proteins associated with G1 progression (E2F1, E2F4 and p107), the differential is in the range of two to four-fold.

## Discussion

### Transcriptional profiles and quantitative immunofluoresence of HMEC reveal significant cancer-associated changes following both selection and immortalization

The effect of malignant transformation (oncogenesis) on gene expression has been studied extensively in both cell lines and tissues in an effort to characterize the causes of cancer at the molecular level [45]. Gene signatures commonly found in breast and other human cancers include those critical for the cell cycle, chromosomal stability and proliferation; the extent of the increase in the expression of this signature correlates with tumor grade and poorer prognosis [26,46]. A separate signature of IFN-regulated genes has also been observed in ductal carcinoma *in situ *(DCIS) [47] and has been associated with metastasis to the lymph nodes in aggressive breast cancers [48]. We have observed both of these signatures in non-malignant, immortally transformed, HMEC lines that had overcome the two senescence barriers to immortalization, despite these lines retaining many characteristics of finite lifespan epithelial cells.

### Transcriptional changes in gene families associated with mammary epithelial biology or breast cancer in post-selection and fully immortalized HMEC

There are several gene families that we identified in this study which have direct connections to breast epithelial biology and breast cancer, which we can summarize:

(A) Several IGFBPs show reduced expression in post-selection HMEC and immortalized lines, including IGFBP2 (minor decreases overall, but larger in the p53^-/- ^lines), *IGFBP3 *and *IGFBP5 *(very large decreases in immortal HMEC). Levels of *IGFBP4 *were significantly reduced in 184B5ME relative to 184B5. IGFBPs are frequently observed to be reduced in breast cancers, and these reductions are associated with increased sensitivity to IGF-I and IGF-II [49,50].

(B) *BRCA1*, a gene deleted in about 5% of women with breast cancer, encodes a protein that interacts with many other proteins [51]. These complexes recognize and orchestrate the repair of DNA damage. Many genes that encode proteins that interact with BRCA1 were identified in this study as genes that increase expression following either selection or immortalization. *BAP, RAD51, CSE1L *and *RFC4 *all increased expression following selection in a pattern similar to the E2F-regulated genes identified as Group C in Figure 3B. *MYC, RAD50 *and *RFC3 *increased expression in fully immortalized lines, including the p53^-/- ^lines. These changes suggest the possibility that BRCA1-mediated functions are affected by overcoming stasis and/or immortalization, which is supported by the significant change in localization of BRCA1 to the nucleus in post-selection HMEC.

(C) The increased expression of a well-characterized cluster of IFN-regulated genes was observed in some lines in this study, as well as in other studies of HMEC [36], and in a taxol-resistant MCF-7 line [52]. The IFN-dependent stress response is mediated by BRCA1 [37,53]. Therefore, since we have noted expression changes in many genes associated with BRCA1 function, as well as in BRCA1 abundance and localization in post-selection HMEC, IFN gene signature may reflect changes in BRCA1-mediated functions.

(D) Inhibitors of Differentiation (ID) genes are important regulators of differentiation by dominantly interfering with the function of bHLH proteins during embryogenesis, neurodevelopment and cancer. Part of their function is through the repression of CKIs, including p16. Some functions have been attributed to specific members, including the interaction of ID2 with Rb [54], and the expression of *BRCA1 *by ID4 [55], which is in turn repressed by BRCA1 [56]. In this study, *ID1 *is expressed at higher levels in the immortalized lines (184AA2 is an exception), while *ID4 *is repressed in post-selection HMEC and all of the immortalized lines.

(E) S100 proteins comprise a large family of calcium-activated proteins that function in homo- and hetero-dimers to regulate many intra- and extra-cellular targets [57]. Their increased expression in cancer and inflammatory diseases has provoked interest in this family as potential drug targets and clinical biomarkers. We observe increases in the expression of *S100A8 *and *S100A9*, which comprise the heterodimer Calprotectin, following selection and further dramatic increases following immortalization. Increased expression of *S100P *is seen in DCIS [58], and was also observed in several of the immortalized lines, particularly 184B5ME, the *ERBB2/Her2 *transduced line. Increased expression of S100A7, also known as psoriasin, is seen in both DCIS and IDC, particularly ER negative breast cancers [59]; increased expression was observed in several immortalized lines, most strongly in 184AA3.

### Transcriptional changes that occur following genetic changes associated with invasive cancer

p53 imposes a cell cycle arrest when chromosomal breakage or damage is detected, and its loss in breast cancer is associated with increased chromosomal instability and a more aggressive subtype [60]. The two *p53*^-/- ^lines we have characterized show a number of transcriptional changes that are expected of *p53*^-/- ^cell lines, as well as changes unique to the two lines. Of note is expression of the IFN-induced genes observed in post-selection 195L cells and in the 184AA3 line. This may indicate a common molecular event occurred following selection of the 195L cells and the immortalization of the 184AA3 cells. Further studies on the changes common and unique to *p53*^-/- ^HMEC lines may be important in understanding differences between *p53*^+/+ ^and *p53*^-/- ^cell lines and breast cancers in overcoming senescence barriers and immortalizing.

In data presented here, transfection of an immortalized line with a clinically-relevant oncogene, *ERBB2/Her2*, showed fewer transcriptional changes than were observed following selection or immortalization, and these changes were generally limited to genes involved in invasive growth and motility. Specifically, expression of the proliferation geneset was not dramatically altered, but there was increased expression of genes encoding the secreted proteases Cystatin E/M, and Kallikrein 6, as well as tissue plasminogen activator. Such changes could enable these cells to grow invasively in breast tissue.

### Activation of transcriptional regulators associated with gene expression changes in post-selection and immortalized HMEC, telomerase reactivation and cancer

In quiescent or unstimulated cells, many transcription factors are excluded from the nucleus and localize to the nucleus upon activation [61]. In the case of BRCA1, nuclear retention has been shown to suppress its pro-apoptotic functions [62]. The proliferation, cell cycle and DNA damage response genes identified in the gene expression signatures we observe are supported by the changes in the localization of several associated regulatory proteins and transcription factors, as determined by quantitative immunofluorescence. Based on previous studies linking regulatory pathways to gene expression, the relationship between the gene expression signatures and the regulatory factor localizations we observe are concordant. Proteins directly responsive to p16/CDK4 activation, particularly Rb, show striking changes in cytoplasmic/nuclear distribution in both post-selection and fully immortalized HMEC, compared to pre-stasis HMEC. Additional proteins also showing strong changes in localization are BRCA1, p53, HDAC3, Myc and SP1. Each of these proteins have well characterized roles in oncogenesis and in the regulation of hTERT [63-66], a critical event in immortalization [1,5]. These changes are consistent with both the transcriptional profiles we have generated of post-selection and fully immortal HMEC, as well as with what is known about the role of these factors on telomerase regulation.

### The relationship between immortalized HMEC and DCIS

Taken together, these data support a classification of immortalized breast epithelial cell lines as *in vitro *models of highly dysregulated epithelial cells, rather than as perpetually growing models of normal breast epithelia. Gene expression patterns we have identified in the comparison of finite-lifespan and immortalized HMEC lines are highly similar to changes observed in DCIS and invasive human breast cancers [47,67,68], and are consistent with other similarities between immortal HMEC lines and DCIS. Specifically, short telomeres and moderate chromosomal instability, as well as telomerase re-activation, are common to many early-stage tumors [69], including the breast [17]. In addition, p16 expression is lost in post-selection, as it is in vHMEC [15,16], which are proposed to be premalignant breast cancer precursors *in vivo*. In contrast, we observe that a cell line, 184B5ME, which grows invasively in tissue culture and in *in vivo *models, shows fewer changes.

DCIS is a complex disease [70], often requiring no immediate treatment in the strict sense, however it is not currently possible to forecast when, or if, progression to IDC will occur. This necessitates an aggressive strategy, even in cases where it may be effectively managed by substantially simpler, cheaper, and less emotionally challenging modes [71]. The ability to characterize DCIS, and to target it explicitly when it manifests invasive potential, is a critical need with regard to effective breast cancer treatment strategies. In particular, established markers for breast cancer, including Ki-67, p53, Her-2^neu ^and ER expression are very effective for identifying aggressive, invasive cancers, and for determining the most effect treatment strategy in these cases, but are less informative about the likelihood that a well-contained DCIS will progress to invasive cancer. Currently, some of the best indicators of DCIS progression risk are cytological, including grade, necrosis and architectural patterns [72]. Additional molecular markers, particularly those that correlate strongly (or better, explain) the histological patterns used to stage DCIS would be very valuable. Some additional molecular markers are emerging. COX-2 has been identified as a marker of vHMEC [15,16], and expression levels have been correlated with DCIS grade, as well [73]. For these reasons, recognizing immortalized HMEC as resembling early-stage cancers would facilitate a formal interrogation of their genetics and physiology for clues to how DCIS occurs, and to the factors that can enable DCIS to progress.

### Use of post-selection and immortalized HMEC to study normal mammary cell biology and breast cancer

Immortalized cell lines have been used to address complex problems in cancer [74] and epithelial cell biology [75] precisely because they allow for controlled experiments to be performed and theories of breast cancer to be tested. In studies of oncogenesis, the non-malignant status of immortalized lines allows for the specific steps in full malignant transformation to be examined, such as by the introduction of activated oncogenes [76,77]. However, in many cases immortalized cell lines are referred to and used as "normal" cells. This inaccurate characterization may obscure understanding of the multiple errors that permit immortal transformation, and thus aspects of early stage carcinogenesis. While established breast cancer cell lines are usually derived from advanced, metastatic tumors (particularly pleural effusions), and therefore are quite different from immortalized cell lines, immortalized lines themselves have undergone extensive genetic and epigenetic changes, especially in frequently studied aspects of oncogenesis, such as G1 checkpoint function and the DNA damage response. The use of immortalized HMEC as "normal" controls for tumor-derived lines can impede our ability to understand early stages of carcinogenesis, and obscure the potential of treating DCIS-stage changes as additional targets for clinical benefit.

## Conclusion

Gene expression profiles and cytological changes in related transcriptional regulators indicate that immortalized HMEC resemble non-invasive breast cancers, such as ductal and lobular carcinomas *in situ*, and are strikingly distinct from finite-lifespan HMEC, particularly with regard to genes involved in proliferation, cell cycle regulation, chromosome structure and the DNA damage response. The comparison of HMEC profiles with lines harboring oncogenic changes (e.g. overexpression of Her-2^neu^, loss of p53 expression) identifies genes involved in tissue remodeling as well as proinflamatory cytokines and S100 proteins. Studies on carcinogenesis using immortalized cell lines as starting points or "normal" controls need to account for the significant pre-existing genetic and epigenetic changes inherent in such lines before results can be broadly interpreted.

## Abbreviations

LI, labeling index; HMEC, human mammary epithelial cells; CKI, cyclin-dependent kinase inhibitor, DCIS, ductal carcinoma in situ; IDC, invasive ductal carcinoma; PD, population doubling; ANOVA, analysis of variance; CIN, chromosomal instability; IGFBP; insulin-like growth factor binding protein; SA-b-gal, senescence associated beta-galactosidase; ECM, extracellular matrix; HCS, high content screening.

## Competing interests

The author(s) declare that they have no competing interests.

## Authors' contributions

JP, JHL, CT, and KS performed experiments and analyzed primary data. YL, J-JL, MW, SJ and SH analyzed normalized data and interpreted results. JG and MS developed cell lines and analyzed normalized data. JP performed cell-based assays on the transcription factors and regulatory proteins. JP and SH analyzed data from the cell-based assays. YL, MS and SH wrote the manuscript. All authors read and approved the final version of the manuscript.

## Supplementary Material

Additional file 1 Table s1: Genes Expressed Concordantly in Pre-Stasis and Post-Selection Cell Types. Compilation of genelists that distinguish the two classes of finite-lifespan HMEC strains. Click here for file

Additional file 2 Table s2. Genes Concordantly Expressed in Pre-stasis, Post-selection or Fully Immortalized HMEC. Compilations of genelists that define the three classes of non-malignant HMEC cell strains and lines. Click here for file

Additional file 3 Table s3: Genes Expressed Concordantly in p53^+/+ ^(184A1 and 184B5) or p53^-/- ^(184AA2 and 184AA3) HMEC. Compilations of commonly expressed genes in multiple wild type and p53^- ^immortalized HMEC cell lines. Click here for file

Additional file 4 Table s4. Gene Expression changes of p53^+ ^cell lines 184A1 versus 184B5. Compilations of genelists and expression statistics of genes expressed uniquely in two p53 wild type HMEC cell lines. Click here for file

Additional file 5 Table s5. Gene Expression changes of p53^+ ^cell lines 184A1 versus 184B5. Compilations of genelists and expression statistics of genes expressed uniquely in two p53^- ^HMEC cell lines. Click here for file

Additional file 6 Table s6. Gene Expression Changes Resulting from Expression of ERB-B2/Her2^neu ^in 184B5. Compilations of genelists and expression statistics of genes that change expression following overexpression of the Her-2^neu ^oncogene. Click here for file
