# Supplementary material for: Transcriptional changes associated with breast cancer occur as normal human mammary epithelial cells overcome senescence barriers and become immortalized
Source: Mol Cancer. 2007 Jan 18;6:7. doi: 10.1186/1476-4598-6-7 (PMC1784108; doi:10.1186/1476-4598-6-7)
Supplement: Additional file 1 — Table s1: Genes Expressed Concordantly in Pre-Stasis and Post-Selection Cell Types. Compilation of genelists that distinguish the two classes of finite-lifespan HMEC strains. [file 1476-4598-6-7-S1.doc]

| Table s1: Genes Expressed Concordantly in Pre-Stasis and Post-Selection Cell Types | |
| --- | --- |
|  | |
| Genes expressed in pre-stasis HMEC | |
|  |  |
| Cytoskeleton |  |
| ACTA2 | actin, alpha 2, smooth muscle, aorta |
| ACTG2 | actin, gamma 2, smooth muscle, enteric |
| CDH2 | cadherin 2, type 1, N-cadherin (neuronal) |
| CNN3 | calponin 3, acidic |
| MACF1 | microtubule-actin crosslinking factor 1 |
| MAP2 | microtubule-associated protein 2 |
| TPM2 | tropomyosin 2 (beta) |
| TUBB | tubulin, beta polypeptide |
|  |  |
| Extracellular Matrix and Cell-Cell Communication |  |
| ADAMTS1 | a disintegrin-like and metalloprotease (reprolysin type)  with thrombospondin type 1 motif, 1 |
| ADAMTS5 | a disintegrin-like and metalloprotease (reprolysin type)  with thrombospondin type 1 motif, 5 (aggrecanase-2) |
| CAST | calpastatin, calpain inhibitor |
| COL1A2 | collagen, type I, alpha 2 |
| COL2A1 | collagen, type II, alpha 1 (primary osteoarthritis,  spondyloepiphyseal dysplasia, congenital) |
| COL4A1 | collagen, type IV, alpha 1 |
| COL6A1 | collagen, type VI, alpha 1 |
| COL6A2 | collagen, type VI, alpha 2 |
| CPA4 | carboxypeptidase A4 |
| CST6 | cystatin E/M |
| CTGF | connective tissue growth factor |
| DSC2 | desmocollin 2 |
| EDIL3 | EGF-like repeats and discoidin I-like domains 3 |
| EGFL5 | EGF-like-domain, multiple 5 |
| ENC1 | ectodermal-neural cortex (with BTB-like domain) |
| ITGB3 | integrin, beta 3 (platelet glycoprotein IIIa, antigen CD61) |
| KLK10 | kallikrein 10 |
| KLK5 | kallikrein 5 |
| KLK6 | kallikrein 6 (neurosin, zyme) |
| KLK7 | kallikrein 7 (chymotryptic, stratum corneum) |
| KRT23 | keratin 23 (histone deacetylase inducible) |
| MCAM | melanoma cell adhesion molecule |
| MGP | matrix Gla protein |
| NET-6 | transmembrane 4 superfamily member tetraspan NET-6 |
| PLOD | procollagen-lysine, 2-oxoglutarate 5-dioxygenase  (lysine hydroxylase, Ehlers-Danlos syndrome type VI) |
| PMP22 | peripheral myelin protein 22 |
| RSN | restin (Reed-Steinberg cell-expressed intermediate  filament-associated protein) |
| TIMP3 | tissue inhibitor of metalloproteinase 3  (Sorsby fundus dystrophy, pseudoinflammatory) |
| TMEPAI | transmembrane, prostate androgen induced RNA |
|  |  |
| Protein Metabolism and Turnover |  |
| CTSB | cathepsin B |
| UCHL1 | ubiquitin carboxyl-terminal esterase L1  (ubiquitin thiolesterase) |
|  |  |
| Protein Secretion |  |
| GCNT1 | glucosaminyl (N-acetyl) transferase 1, core 2  (beta-1,6-N-acetylglucosaminyltransferase) |
| TRAM | translocating chain-associating membrane protein |
|  |  |
| Metabolism and Homeostasis |  |
| BPGM | 2,3-bisphosphoglycerate mutase |
| CLIC3 | chloride intracellular channel 3 |
| LIPG | lipase, endothelial |
| LOXL2 | lysyl oxidase-like 2 |
| SLC16A4 | solute carrier family 16 (monocarboxylic acid transporters)  , member 4 |
| SLC17A5 | solute carrier family 17 (anion/sugar transporter),  member 5 |
|  |  |
| Transcription and Translation |  |
| GLIS2 | Kruppel-like zinc finger protein GLIS2 |
| IRX1 | iroquois homeobox protein 1 |
|  |  |
| Signal Transduction |  |
| OXTR | oxytocin receptor |
| RSU1 | Ras suppressor protein 1 |
| STAC | src homology three (SH3) and cysteine rich domain |
| SORT1 | sortilin 1; NTr co-receptor for nerve growth factor |
|  |  |
| Cell Cycle |  |
| CDKN2B | cyclin-dependent kinase inhibitor 2B (p15, inhibits CDK4) |
|  |  |
| Other |  |
| DESC1 | DESC1 protein |
| DKFZP564G202 | DKFZP564G202 protein |
| FLJ11036 | hypothetical protein FLJ11036 |
| FLJ40021 | hypothetical protein FLJ40021 |
| KIAA0275 | KIAA0275 gene product |
| KIAA1497 | KIAA1497 protein |
| LBH | likely ortholog of mouse limb-bud and heart gene |
| LOC115207 | hypothetical protein BC013764 |
| LOC91663 | hypothetical protein BC013995 |
| LOC92689 | hypothetical protein BC001096 |
| MIG2 | mitogen inducible 2 |
| PPAP2A | phosphatidic acid phosphatase type 2A |
| SPG3A | spastic paraplegia 3A (autosomal dominant) |
| TRIM2 | tripartite motif-containing 2 |
| WSB2 | likely ortholog of mouse WD-40-repeat-containing  protein with a SOCS box 2 |
|  |  |
| Genes expressed in post-selection HMEC | |
|  |  |
| Extracellular Matrix and Cell-Cell Communication |  |
| CSPG2 | chondroitin sulfate proteoglycan 2 (versican) |
| DLG7 | discs, large homolog 7 (Drosophila) |
| IFI16 | interferon, gamma-inducible protein 16 |
| IL18 | interleukin 18 (interferon-gamma-inducing factor) |
| IL1A | interleukin 1, alpha |
| ITGB3BP | integrin beta 3 binding protein (beta3-endonexin) |
| HBP17 | heparin-binding growth factor binding protein |
| PLAB | prostate differentiation factor; GDF15 |
| PMSCL1 | polymyositis/scleroderma autoantigen 1, 75kDa |
| PLAU | plasminogen activator, urokinase |
| S100A8 | S100 calcium binding protein A8 (calgranulin A) |
| SERPINA1 | serine (or cysteine) proteinase inhibitor, clade A  (alpha-1 antiproteinase, antitrypsin), member 1 |
| SERPINB3 | serine (or cysteine) proteinase inhibitor, clade B  (ovalbumin), member 3 |
|  |  |
| Protein Metabolism and Turnover |  |
| HSPC150 | HSPC150 protein similar to ubiquitin-conjugating enzyme |
| PSMB8 | proteasome (prosome, macropain) subunit, beta type,  8 (large multifunctional protease 7) |
| UBE2C | ubiquitin-conjugating enzyme E2C |
| UHRF1 | ubiquitin-like, containing PHD and RING finger domains, 1 |
|  |  |
| Metabolism and Homeostasis |  |
| DHFR | dihydrofolate reductase |
| PAICS | phosphoribosylaminoimidazole carboxylase,  phosphoribosylaminoimidazole succinocarboxamide  synthetase |
|  |  |
| Transcription and Translation |  |
| ANKT | nucleolar protein ANKT |
| LYAR | hypothetical protein FLJ20425 |
| MKI67 | antigen identified by monoclonal antibody Ki-67 |
| RAMP | RA-regulated nuclear matrix-associated protein |
|  |  |
| Signal Transduction |  |
| GG2-1 | TNF-induced protein; TNFAIP8, oncogenic negative  regulator of extrinsic apoptosis |
| LGN | LGN protein; GPSM2, regulator of heterotrimeric  G-protein signaling |
| MELK | maternal embryonic leucine zipper kinase |
| SHCBP1 | likely ortholog of mouse Shc SH2-domain binding  protein 1 |
| TOPK | T-LAK cell-originated protein kinase |
| VRP | vascular Rab-GAP/TBC-containing |
|  |  |
| Cell Cycle |  |
| BIRC5 | baculoviral IAP repeat-containing 5 (survivin) |
| BUB1 | BUB1 budding uninhibited by benzimidazoles 1  homolog (yeast) |
| BUB3 | BUB3 budding uninhibited by benzimidazoles 3  homolog (yeast) |
| CCNA2 | cyclin A2 |
| CCNB1 | cyclin B1 |
| CCNB2 | cyclin B2 |
| CDC2 | cell division cycle 2, G1 to S and G2 to M |
| CDC25B | cell division cycle 25B |
| CDKN3 | cyclin-dependent kinase inhibitor 3 (CDK2-associated  dual specificity phosphatase) |
| CKS1B | CDC28 protein kinase regulatory subunit 1B |
| CSE1L | CSE1 chromosome segregation 1-like (yeast) |
| GMNN | geminin, DNA replication inhibitor |
| MAD2L1 | MAD2 mitotic arrest deficient-like 1 (yeast) |
| MCM2 | MCM2 minichromosome maintenance deficient 2,  mitotin (S. cerevisiae) |
| MCM6 | MCM6 minichromosome maintenance deficient 6  (MIS5 homolog, S. pombe) (S. cerevisiae) |
| MCM7 | MCM7 minichromosome maintenance deficient 7  (S. cerevisiae) |
| NASP | nuclear autoantigenic sperm protein (histone-binding) |
| PCNA | proliferating cell nuclear antigen |
| PRC1 | protein regulator of cytokinesis 1 |
| RFC4 | replication factor C (activator 1) 4, 37kDa |
| RRM2 | ribonucleotide reductase M2 polypeptide |
| SMC4L1 | SMC4 structural maintenance of chromosomes  4-like 1 (yeast) |
| STK12 | serine/threonine kinase 12 |
| TOP2A | topoisomerase (DNA) II alpha 170kDa |
| ZWINT | ZW10 interactor |
|  |  |
| Other |  |
| C10orf3 | chromosome 10 open reading frame 3 |
| C20orf1 | chromosome 20 open reading frame 1 |
| CMG2 | capillary morphogenesis protein 2 |
| DKFZp762E1312 | hypothetical protein DKFZp762E1312 |
| FLJ20354 | hypothetical protein FLJ20354 |
| HMGB2 | high-mobility group box 2 |
| HMGN2 | high-mobility group nucleosomal binding domain 2 |
| IER5 | immediate early response 5 |
| KIAA0101 | KIAA0101 gene product |
| KIAA0186 | KIAA0186 gene product |
| KIAA1393 | KIAA1393 protein |
| LOC113115 | hypothetical protein BC011716 |
| LOC134147 | hypothetical protein BC001573 |
| LOC51659 | HSPC037 protein |
| MGC34923 | hypothetical protein MGC34923 |
| PRO2000 | PRO2000 protein |
| PSIP2 | PC4 and SFRS1 interacting protein 2 |
| SRPX | sushi-repeat-containing protein, X chromosome |
