# Supplementary material for: Transcriptional changes associated with breast cancer occur as normal human mammary epithelial cells overcome senescence barriers and become immortalized
Source: Mol Cancer. 2007 Jan 18;6:7. doi: 10.1186/1476-4598-6-7 (PMC1784108; doi:10.1186/1476-4598-6-7)
Supplement: Additional file 2 — Table s2. Genes Concordantly Expressed in Pre-stasis, Post-selection or Fully Immortalized HMEC. Compilations of genelists that define the three classes of non-malignant HMEC cell strains and lines. [file 1476-4598-6-7-S2.doc]

| Table s2. Genes Concordantly Expressed in Pre-stasis, Post-selection or Fully Immortalized HMEC | | |  |
| --- | --- | --- | --- |
|  | | |  |
|  | | |  |
| Genes over-expressed in pre-stasis HMEC | | |  |
|  | | |  |
| Cytoskeleton | |  |  |
| ACTA2 | | actin, alpha 2, smooth muscle, aorta |  |
| ACTG2 | | actin, gamma 2, smooth muscle, enteric |  |
| CDH2 | | cadherin 2, type 1, N-cadherin (neuronal) |  |
| MAP2 | | microtubule-associated protein 2 |  |
| RSN | | restin (Reed-Steinberg cell-expressed intermediate filament-  associated protein) |  |
| TPM2 | | tropomyosin 2 (beta) |  |
| Extracellular Matrix and Cell-Cell Interactions | | |  |
| ADAMTS5 | | a disintegrin-like and metalloprotease (reprolysin type)  with thrombospondin type 1 motif, 5 (aggrecanase-2) |  |
| COL1A2 | | collagen, type I, alpha 2 |  |
| COL2A1 | | collagen, type II, alpha 1 (primary osteoarthritis, spondyloepiphyseal  dysplasia, congenital) |  |
| COL4A1 | | collagen, type IV, alpha 1 |  |
| COL4A2 | | collagen, type IV, alpha 2 |  |
| COL6A1 | | collagen, type VI, alpha 1 |  |
| COL6A2 | | collagen, type VI, alpha 2 |  |
| CST6 | | cystatin E/M |  |
| CTSB | | cathepsin B |  |
| DSC2 | | desmocollin 2 |  |
| EGFL5 | | EGF-like-domain, multiple 5 |  |
| GJA5 | | gap junction protein, alpha 5, 40kDa (connexin 40) |  |
| IGFBP3 | | insulin-like growth factor binding protein 3 |  |
| ITGB3 | | integrin, beta 3 (platelet glycoprotein IIIa, antigen CD61) |  |
| KLK6 | | kallikrein 6 (neurosin, zyme) |  |
| KLK7 | | kallikrein 7 (chymotryptic, stratum corneum) |  |
| KLK10 | | kallikrein 10 |  |
| KRT23 | | keratin 23 (histone deacetylase inducible) |  |
| LIPG | | lipase, endothelial |  |
| LOC143903 | | layilin |  |
| LTBP2 | | latent transforming growth factor beta binding protein 2 |  |
| MGP | | matrix Gla protein |  |
| MIG2 | | mitogen inducible 2 |  |
| MOX2 | | antigen identified by monoclonal antibody MRC OX-2 |  |
| NET-6 | | transmembrane 4 superfamily member tetraspan NET-6 |  |
| NY-REN-25 | | NY-REN-25 antigen |  |
| PMP22 | | peripheral myelin protein 22 |  |
| SERPING1 | | serine (or cysteine) proteinase inhibitor, clade G (C1 inhibitor),  member 1, (angioedema, hereditary) |  |
| SPP1 | | secreted phosphoprotein 1 (osteopontin, bone sialoprotein I,  early T-lymphocyte activation 1) |  |
| TIMP3 | | tissue inhibitor of metalloproteinase 3 (Sorsby fundus dystrophy,  pseudoinflammatory) |  |
| WNT5B | | wingless-type MMTV integration site family, member 5B |  |
|  | |  |  |
| Metabolism | |  |  |
| BPGM | | 2,3-bisphosphoglycerate mutase |  |
| CLIC3 | | chloride intracellular channel 3 |  |
| FADS3 | | fatty acid desaturase 3 |  |
| SLC16A4 | | solute carrier family 16 (monocarboxylic acid transporters),  member 4 |  |
|  | |  |  |
| Protein Biogenesis and Turnover | | |  |
| SELM | | selenoprotein SelM |  |
| UCHL1 | | ubiquitin carboxyl-terminal esterase L1 (ubiquitin thiolesterase) |  |
|  | |  |  |
| Protein Secretion | |  |  |
| COPZ2 | | coatomer protein complex, subunit zeta 2 |  |
| DESC1 | | DESC1 protein |  |
| EHD3 | | EH-domain containing 3 |  |
| GCNT1 | | glucosaminyl (N-acetyl) transferase 1, core 2 (beta-  1,6-N-acetylglucosaminyltransferase) |  |
| SEC14L2 | | SEC14-like 2 (S. cerevisiae) |  |
| SORT1 | | sortilin 1 |  |
| TRAM | | translocating chain-associating membrane protein |  |
|  | |  |  |
| Signal Transduction | |  |  |
| CHRNB1 | | cholinergic receptor, nicotinic, beta polypeptide 1 (muscle) |  |
| OXTR | | oxytocin receptor |  |
|  | |  |  |
| Transcription and Translation | | |  |
| GLIS2 | | Kruppel-like zinc finger protein GLIS2 |  |
| LMCD1 | | LIM and cysteine-rich domains 1 |  |
| RBP1 | | retinol binding protein 1, cellular |  |
| RUNX1 | | runt-related transcription factor 1 (acute myeloid leukemia 1;  aml1 oncogene) |  |
|  | |  |  |
| Other | |  |  |
| AF1Q | | ALL1-fused gene from chromosome 1q |  |
| ALEX2 | | armadillo repeat protein ALEX2 |  |
| DESC1 | | DESC1 protein |  |
| DKFZP564G202 | | DKFZP564G202 protein |  |
| DKFZP586H2123 | | DKFZP586H2123 protein |  |
| FLJ14054 | | hypothetical protein FLJ14054 |  |
| FLJ40021 | | hypothetical protein FLJ40021 |  |
| KIAA0275 | | KIAA0275 gene product |  |
| KIAA0599 | | KIAA0599 protein |  |
| KIAA1161 | | KIAA1161 protein |  |
| KIAA1497 | | KIAA1497 protein |  |
|  | |  |  |
| Genes over-expressed in post-selection HMEC | | |  |
|  | |  |  |
| Cytoskeleton | |  |  |
| ACTN1 | | actinin, alpha 1 |  |
| ACTN4 | | actinin, alpha 4 |  |
| KIAA0992 | | palladin, interacts with a-actinin |  |
| NID | | nidogen (enactin) |  |
| TAGLN | | transgelin |  |
| SRPX | | sushi-repeat-containing protein, X chromosome |  |
| TPM4 | | tropomyosin 4 |  |
| BEX1 | | brain expressed, X-linked 1 |  |
| C20orf80, CRIP2 | | chromosome 20 open reading frame 80, cysteine-rich protein 2 |  |
| P311 | | P311 protein |  |
|  | |  |  |
| Extracellular Matrix and Cell-Cell Interactions | | |  |
| ADAM23 | | a disintegrin and metalloproteinase domain 23 |  |
| ADAMTS1 | | a disintegrin-like and metalloprotease (reprolysin type) with thrombospondin type 1 motif, 1 |  |
| CMG2 | | capillary morphogenesis protein 2, anthrax co-receptor with TEM-8 |  |
| CNTN1 | | contactin 1 |  |
| CNTN3 | | contactin 3 (plasmacytoma associated) |  |
| COL5A2 | | collagen, type V, alpha 2 |  |
| CSPG2 | | chondroitin sulfate proteoglycan 2 (versican) |  |
| CTGF | | connective tissue growth factor |  |
| DRAPC1 | | hypothetical protein DRAPC1, regulated be b-catenin |  |
| EDIL3 | | EGF-like repeats and discoidin I-like domains 3 |  |
| FN1 | | fibronectin 1 |  |
| FZD7 | | frizzled homolog 7 (Drosophila) |  |
| GJB2 | | gap junction protein, beta 2, 26kDa (connexin 26) |  |
| HNT | | neurotrimin |  |
| ITGB6 | | integrin, beta 6 |  |
| KRT6B | | keratin 6B |  |
| L1CAM | | L1 cell adhesion molecule (hydrocephalus, stenosis of aqueduct of Sylvius 1, MASA (mental retardation, aphasia, shuffling gait and adducted thumbs) syndrome, spastic paraplegia 1) |  |
| MIA | | melanoma inhibitory activity |  |
| MMP10 | | matrix metalloproteinase 10 (stromelysin 2) |  |
| MMP14 | | matrix metalloproteinase 14 (membrane-inserted) |  |
| MMP2 | | matrix metalloproteinase 2 (gelatinase A, 72kDa gelatinase, 72kDa type IV collagenase) |  |
| MPPE1 | | metallo phosphoesterase |  |
| PCDH19 | | protocadherin 19 |  |
| PLAU | | plasminogen activator, urokinase |  |
| PTHLH | | parathyroid hormone-like hormone |  |
| SERPINA1 | | serine (or cysteine) proteinase inhibitor, clade A (alpha-1 antiproteinase, antitrypsin), member 1 |  |
| SERPINE2 | | serine (or cysteine) proteinase inhibitor, clade E (nexin, plasminogen activator inhibitor type 1), member 2 |  |
| TMEM2 | | transmembrane protein 2 |  |
| TRA1 | | tumor rejection antigen (gp96) 1 |  |
| TRAP150 | | thyroid hormone receptor-associated protein, 150 kDa subunit |  |
| TTC3 | | tetratricopeptide repeat domain 3 |  |
| VLDLR | | very low density lipoprotein receptor |  |
|  | |  |  |
| Metabolism | |  |  |
| ATP1B1 | | ATPase, Na+/K+ transporting, beta 1 polypeptide |  |
| CAT | | catalase |  |
| CLN2 | | ceroid-lipofuscinosis, neuronal 2, late infantile (Jansky-Bielschowsky disease) |  |
| GCLM | | glutamate-cysteine ligase, modifier subunit |  |
| HEPH | | hephaestin; iron homeostasis, macular degenration linked |  |
| KMO | | kynurenine 3-monooxygenase (kynurenine 3-hydroxylase) |  |
| SLC7A8 | | solute carrier family 7 (cationic amino acid transporter, y+ system), member 8 |  |
|  | |  |  |
| Protein Biogenesis and Turnover | | |  |
| BHLHB3 | | basic helix-loop-helix domain containing, class B, 3; lysosomal protease |  |
| CTSB | | cathepsin B |  |
| CTSL2 | | cathepsin L2 |  |
| PA200 | | proteasome activator 200 kDa |  |
| TPST1 | | tyrosylprotein sulfotransferase 1 |  |
|  | |  |  |
| Protein Secretion | |  |  |
| CALU | | calumenin |  |
| LPHH1 | | latrophilin 1 |  |
|  | |  |  |
| Signal Transduction | |  |  |
| ARK5 | | KIAA0537 gene product, IGF-1 signaling, metastasis and invasion of myeloma cells |  |
| INHBA | | inhibin, beta A (activin A, activin AB alpha polypeptide) |  |
| IQGAP1 | | IQ motif containing GTPase activating protein 1 |  |
| MYLK | | myosin, light polypeptide kinase |  |
| PTK7 | | PTK7 protein tyrosine kinase 7 |  |
| SPRY2 | | sprouty homolog 2 (Drosophila), neg regul EGFR signaling, dn-reg in PrCa |  |
|  | |  |  |
| Transcription and Translation | | |  |
| HDAC3 | | histone deacetylase 3 |  |
|  | |  |  |
| Other | |  |  |
| C20orf80, CRIP2 | | chromosome 20 open reading frame 80, cysteine-rich protein 2 |  |
| DKFZP564K0322 | | hypothetical protein DKFZp564K0322 |  |
| DKFZp564O1278,  FLJ22774 | | hypothetical protein DKFZp564O1278, hypothetical protein FLJ22774 |  |
| DKFZP761F241 | | hypothetical protein DKFZp761F241 |  |
| FLJ10856 | | hypothetical protein FLJ10856 |  |
| FLJ20481 | | hypothetical protein FLJ20481 |  |
| FLJ31810 | | hypothetical protein FLJ31810 |  |
| FLJ90440 | | hypothetical protein FLJ90440 |  |
| MGC12335 | | hypothetical protein MGC12335 |  |
|  | |  |  |
| Genes over-expressed in fully immortalized HMEC | | |  |
|  | |  |  |
| Cytoskeleton | | |  |
| STOML2 | stomatin (EPB72)-like 2; raft assocation | |  |
|  | | |  |
| ECM and Cell-Cell Communication | | |  |
| 8D6A | | 8D6 antigen |  |
| C1QBP, MGC4189 | | complement component 1, q subcomponent binding protein, hypothetical protein MGC4189 |  |
| IL18 | | interleukin 18 (interferon-gamma-inducing factor) |  |
| LRPPRC | | leucine-rich PPR-motif containing |  |
| NMU | | neuromedin U |  |
| PDZK3 | | PDZ domain containing 3 |  |
| PTGES | | prostaglandin E synthase; PIG12, p53-induced |  |
| SECTM1 | | secreted and transmembrane 1 |  |
| HDGF | | hepatoma-derived growth factor (high-mobility group protein 1-like) |  |
|  | |  |  |
| Metabolism and Homeostasis | | |  |
| ADA | | adenosine deaminase |  |
| ADPRT | | ADP-ribosyltransferase (NAD+; poly (ADP-ribose) polymerase); PARP |  |
| ATP5O | | ATP synthase, H+ transporting, mitochondrial F1 complex, O subunit (oligomycin sensitivity conferring protein) |  |
| CLNS1A | | chloride channel, nucleotide-sensitive, 1A |  |
| CYC1 | | cytochrome c-1 |  |
| DC12, SCNN1A | | DC12 protein, sodium channel, nonvoltage-gated 1 alpha |  |
| EEG1 | | likely ortholog of mouse embryonic epithelial gene 1, transporter |  |
| IMPDH2 | | IMP (inosine monophosphate) dehydrogenase 2 |  |
| KYNU | | kynureninase (L-kynurenine hydrolase) |  |
| MAOA | | monoamine oxidase A |  |
| MCCC2 | | methylcrotonoyl-Coenzyme A carboxylase 2 (beta) |  |
| MFTC | | mitochondrial folate transporter/carrier |  |
| OXA1L | | oxidase (cytochrome c) assembly 1-like |  |
| SDHB | | succinate dehydrogenase complex, subunit B, iron sulfur (Ip) |  |
| SLC21A12 | | solute carrier family 21 (organic anion transporter), member 12 |  |
| SORD | | sorbitol dehydrogenase |  |
| SUCLG1 | | succinate-CoA ligase, GDP-forming, alpha subunit |  |
|  | |  |  |
| Protein Biogenesis and Turnover | | |  |
| CABC1 | | chaperone, ABC1 activity of bc1 complex like (S. pombe) |  |
| HS6ST2 | | heparan sulfate 6-O-sulfotransferase 2 |  |
| HSPA9B | | heat shock 70kDa protein 9B (mortalin-2) |  |
| HSPD1 | | heat shock 60kDa protein 1 (chaperonin) |  |
| PPT1 | | palmitoyl-protein thioesterase 1 (ceroid-lipofuscinosis, neuronal 1, infantile) |  |
| TRAP1 | | heat shock protein 75 |  |
| USP3 | | ubiquitin specific protease 3 |  |
|  | |  |  |
| Protein Secretion | |  |  |
| MAL2 | | mal, T-cell differentiation protein 2 |  |
|  | |  |  |
| Signal Transduction | |  |  |
| ADRB2 | | adrenergic, beta-2-, receptor, surface |  |
| DDEF1 | | development and differentiation enhancing factor 1, ARF GAP |  |
| DDX18 | | DEAD/H (Asp-Glu-Ala-Asp/His) box polypeptide 18 (Myc-regulated) |  |
| FKBP5 | | FK506 binding protein 5 |  |
| VIP32 | | hypothetical protein PP5395, activator of MAPK signaling |  |
|  | |  |  |
| Cell Cycle | |  |  |
| CDC25B | | cell division cycle 25B |  |
| CDCA7 | | cell division cycle associated 7 |  |
| CKS1B | | CDC28 protein kinase regulatory subunit 1B |  |
| NEK2 | | NIMA (never in mitosis gene a)-related kinase 2 |  |
| STK6 | | serine/threonine kinase 6 |  |
|  | |  |  |
| Transcription and Translation | | |  |
| DKC1 | | dyskeratosis congenita 1, dyskerin; ribosomal function, binds TERC |  |
| DSIPI | | delta sleep inducing peptide, immunoreactor; GILZ, IL-10 induced, antiinflamatory and antiapoptotic role |  |
| EEF1D | | eukaryotic translation elongation factor 1 delta (guanine nucleotide exchange protein) |  |
| EIF3S6 | | eukaryotic translation initiation factor 3, subunit 6 48kDa |  |
| FBL | | fibrillarin; nucleolar protein required for rRNA processing |  |
| FTSJ2 | | FtsJ homolog 2 (E. coli), nucleolar rRNA methyl-transferase |  |
| GEMIN5 | | gem (nuclear organelle) associated protein 5 |  |
| ID3 | | inhibitor of DNA binding 3, dominant negative helix-loop-helix protein |  |
| KARS | | lysyl-tRNA synthetase |  |
| MAGOH | | mago-nashi homolog, proliferation-associated (Drosophila); nucleolar exon-junction complex protein |  |
| MRPL22 | | mitochondrial ribosomal protein L22 |  |
| MRPL3 | | mitochondrial ribosomal protein L3 |  |
| MRPL30 | | mitochondrial ribosomal protein L30 |  |
| MRPS27 | | mitochondrial ribosomal protein S27 |  |
| MYC | | v-myc myelocytomatosis viral oncogene homolog (avian) |  |
| NOL5A | | nucleolar protein 5A (56kDa with KKE/D repeat) |  |
| NOLA2 | | nucleolar protein family A, member 2 (H/ACA small nucleolar RNPs) |  |
| NOLC1 | | nucleolar and coiled-body phosphoprotein 1 |  |
| NUP133 | | nucleoporin 133kDa |  |
| PRPF4 | | PRP4 pre-mRNA processing factor 4 homolog (yeast) |  |
| RFC4 | | replication factor C (activator 1) 4, 37kDa |  |
| RIP60 | | replication initiation region protein (60kD) |  |
| RPC5 | | RNA polymerase III 80 kDa subunit RPC5 |  |
| Rpo1-2 | | similar to DNA-directed RNA polymerase I (135 kDa) |  |
| RPS21 | | ribosomal protein S21 |  |
| SNX5 | | sorting nexin 5 |  |
| TCERG1 | | transcription elongation regulator 1 (CA150) |  |
| TCOF1 | | Treacher Collins-Franceschetti syndrome 1; pre-rRNA methylation, neural crest and macular degeneration linked |  |
| WDR3 | | WD repeat domain 3 |  |
| ZRF1 | | zuotin related factor 1; MPHOSH11, ribosomal co-chaparone |  |
|  | |  |  |
| Other | |  |  |
| C20orf44 | | chromosome 20 open reading frame 44 |  |
| CECR5 | | cat eye syndrome chromosome region, candidate 5, CHR22 |  |
| CGI-09 | | CGI-09 protein |  |
| DKFZP564M182 | | DKFZP564M182 protein |  |
| DKFZp762L0311 | | hypothetical protein DKFZp762L0311 |  |
| FLJ10407 | | hypothetical protein FLJ10407 |  |
| FLJ10439 | | hypothetical protein FLJ10439 |  |
| FLJ12436 | | hypothetical protein FLJ12436 |  |
| JTB | | jumping translocation breakpoint |  |
| MTX1 | | metaxin 1 |  |
| TH1L | | TH1-like (Drosophila) |  |
