# Supplementary material for: Transcriptional changes associated with breast cancer occur as normal human mammary epithelial cells overcome senescence barriers and become immortalized
Source: Mol Cancer. 2007 Jan 18;6:7. doi: 10.1186/1476-4598-6-7 (PMC1784108; doi:10.1186/1476-4598-6-7)
Supplement: Additional file 3 — Table s3: Genes Expressed Concordantly in p53+/+ (184A1 and 184B5) or p53-/- (184AA2 and 184AA3) HMEC. Compilations of commonly expressed genes in multiple wild type and p53- immortalized HMEC cell lines. [file 1476-4598-6-7-S3.doc]

| **Table s3: Genes Expressed Concordantly in p53^+/+^ (184A1 and 184B5) or p53^-/-^ (184AA2 and 184AA3) HMEC.** | |
| --- | --- |
|  |  |
| Genes overexpressed in p53^+/+^ HMEC lines over p53^-/-^ HMEC lines | |
|  |  |
| Cytoskeleton |  |
| CYFIP2 | cytoplasmic FMR1 interacting protein 2 |
| EMS1 | ems1 sequence (mammary tumor and squamous cell carcinoma-associated (p80/85 src substrate); cortactin, part of 11q13 amplicon |
| FAT | FAT tumor suppressor homolog 1 (Drosophila) |
| FLNB | filamin B, beta (actin binding protein 278) |
| RAI14 | retinoic acid induced 14 |
| TRIM22 | tripartite motif-containing 22 |
| TRIM5 | tripartite motif-containing 5 |
| TUBA3 | tubulin, alpha 3 |
| TUBB-5 | tubulin beta-5 |
| WDR1 | WD repeat domain 1 |
|  |  |
| ECM and Cell-Cell Communication | |
| ADM | adrenomedullin |
| BST2 | bone marrow stromal cell antigen 2 |
| CD59 | CD59 antigen p18-20 (antigen identified by monoclonal antibodies 16.3A5, EJ16, EJ30, EL32 and G344) |
| COL12A1 | collagen, type XII, alpha 1 |
| FAP | fibroblast activation protein, alpha |
| FBN1 | fibrillin 1 (Marfan syndrome) |
| FBN2 | fibrillin 2 (congenital contractural arachnodactyly) |
| FLRT3 | fibronectin leucine rich transmembrane protein 3 |
| FN1 | fibronectin 1 |
| FSTL1 | follistatin-like 1 |
| HAS3 | hyaluronan synthase 3 |
| HBP17 | heparin-binding growth factor binding protein |
| HSPG2 | heparan sulfate proteoglycan 2 (perlecan) |
| IFITM1, PTS | 6-pyruvoyltetrahydropterin synthase, interferon induced transmembrane protein 1 (9-27) |
| IGFBP6 | insulin-like growth factor binding protein 6 |
| IGSF4 | immunoglobulin superfamily, member 4 |
| ITGB5 | integrin, beta 5 |
| KIAA1260 | neuroligin |
| KRT15 | keratin 15 |
| LIF | leukemia inhibitory factor (cholinergic differentiation factor) |
| LTBP2 | latent transforming growth factor beta binding protein 2 |
| LTBP3 | latent transforming growth factor beta binding protein 3 |
| NRCAM | neuronal cell adhesion molecule |
| PLAC8 | placenta-specific 8 |
| PMP22 | peripheral myelin protein 22 |
| SDCCAG8 | serologically defined colon cancer antigen 8 |
| SRI | sorcin |
| SYT8 | synaptotagmin VIII |
| TEM6 | tumor endothelial marker 6 |
| TIMP3 | tissue inhibitor of metalloproteinase 3 (Sorsby fundus dystrophy, pseudoinflammatory) |
| TMEM2 | transmembrane protein 2 |
| TMEPAI | transmembrane, prostate androgen induced RNA |
| TSPAN-1 | tetraspan 1 |
|  |  |
| Metabolism and Homeostasis | |
| ADH5 | alcohol dehydrogenase 5 (class III), chi polypeptide |
| ALDH1A3 | aldehyde dehydrogenase 1 family, member A3 |
| ALDH3A1 | aldehyde dehydrogenase 3 family, memberA1 |
| ARL7 | ADP-ribosylation factor-like 7 |
| DPYSL4 | dihydropyrimidinase-like 4 |
| FLJ23462 | duodenal cytochrome b |
| FXYD3 | FXYD domain containing ion transport regulator 3 |
| FXYD5 | FXYD domain containing ion transport regulator 5 |
| GABRE | gamma-aminobutyric acid (GABA) A receptor, epsilon |
| GAMT | guanidinoacetate N-methyltransferase |
| GYS1 | glycogen synthase 1 (muscle) |
| INPP5D | inositol polyphosphate-5-phosphatase, 145kDa |
| KCNJ15 | potassium inwardly-rectifying channel, subfamily J, member 15 |
| KCNN4 | potassium intermediate/small conductance calcium-activated channel, subfamily N, member 4 |
| PKD2 | polycystic kidney disease 2 (autosomal dominant) |
|  |  |
| Protein Biogenesis and Turnover | |
| CASP6 | caspase 6, apoptosis-related cysteine protease |
| CAST | calpastatin |
| CTSD | cathepsin D (lysosomal aspartyl protease) |
| CXX1 | CAAX box 1 |
| FBXL2 | F-box and leucine-rich repeat protein 2 |
| LAMP2 | lysosomal-associated membrane protein 2 |
| MIPEP | mitochondrial intermediate peptidase |
| MSRA | methionine sulfoxide reductase A |
| NEDD4L | neural precursor cell expressed, developmentally down-regulated 4-like |
| PSMC2 | proteasome (prosome, macropain) 26S subunit, ATPase, 2 |
| SPG3A | spastic paraplegia 3A (autosomal dominant) |
|  |  |
| Protein Secretion |  |
| CPA4 | carboxypeptidase A4 |
| GALNT1 | UDP-N-acetyl-alpha-D-galactosamine:polypeptide N-acetylgalactosaminyltransferase 1 (GalNAc-T1) |
| N33 | Putative prostate cancer tumor suppressor |
| SEC14L2 | SEC14-like 2 (S. cerevisiae) |
| SULF2. | similar to glucosamine-6-sulfatases |
| VAMP8 | vesicle-associated membrane protein 8 (endobrevin) |
|  |  |
| Signal Transduction |  |
| ADORA2B | adenosine A2b receptor |
| ARHGDIB | Rho GDP dissociation inhibitor (GDI) beta |
| ANKRD3 | ankyrin repeat domain 3; PKCd-interacting protein kinase |
| COPS3 | COP9 constitutive photomorphogenic homolog subunit 3 (Arabidopsis) |
| DXS1283E | GS2 gene; phospholipase A2 |
| FYN | FYN oncogene related to SRC, FGR, YES |
| EPS8R2 | EPS8-related protein 2; EGFR substrate |
| G1P3 | interferon, alpha-inducible protein (clone IFI-6-16) |
| GPR48 | G protein-coupled receptor 48 |
| GRP58 | glucose regulated protein, 58kDa |
| HRASLS3 | HRAS-like suppressor 3 |
| NOTCH1 | Notch homolog 1, translocation-associated (Drosophila) |
| PIK3C3 | phosphoinositide-3-kinase, class 3 |
| PSTPIP2 | proline-serine-threonine phosphatase interacting protein 2 |
| SH3BGRL | SH3 domain binding glutamic acid-rich protein like |
|  |  |
| Transcription and Translation | |
| CSTF1 | cleavage stimulation factor, 3' pre-RNA, subunit 1, 50kDa |
| HNRPDL | heterogeneous nuclear ribonucleoprotein D-like |
| HOXC10 | homeo box C10 |
| PCNA | proliferating cell nuclear antigen |
| PCNP, POLR2L | PEST-containing nuclear protein, polymerase (RNA) II (DNA directed) polypeptide L, 7.6kDa |
| RBM3 | RNA binding motif protein 3 |
| RBM8A | RNA binding motif protein 8A |
| RBPMS | RNA-binding protein gene with multiple splicing |
| RPL28 | ribosomal protein L28 |
| RPS27L | ribosomal protein S27-like |
| SFRS11 | splicing factor, arginine/serine-rich 11 |
| TP53 | tumor protein p53 (Li-Fraumeni syndrome) |
| ZDHHC2 | zinc finger, DHHC domain containing 2 |
| ZIC2 | Zic family member 2 (odd-paired homolog, Drosophila); TF involved in brain development |
| ZNF195 | zinc finger protein 195 |
|  |  |
| Cell Cycle and DNA Replication | |
| CDT1 | DNA replication factor |
| RRM2 | ribonucleotide reductase M2 polypeptide |
|  |  |
| Other |  |
| APXL | apical protein-like (Xenopus laevis) |
| BNIP3L | BCL2/adenovirus E1B 19kDa interacting protein 3-like; proapoptotic mitochondial BH3 protein |
| BTBD3 | BTB (POZ) domain containing 3 |
| C11orf13 | chromosome 11 open reading frame 13 |
| C7orf10 | chromosome 7 open reading frame 10 |
| CGI-125 | CGI-125 protein |
| CGI-145 | CGI-145 protein |
| D4S234E | DNA segment on chromosome 4 (unique) 234 expressed sequence |
| DKFZp434L142 | hypothetical protein DKFZp434L142 |
| DKFZP564K1964 | DKFZP564K1964 protein |
| DKFZp564O1278,  FLJ22774 | hypothetical protein DKFZp564O1278, hypothetical protein FLJ22774 |
| DKFZp762E1312 | hypothetical protein DKFZp762E1312 |
| FLJ12436 | hypothetical protein FLJ12436 |
| FLJ21313 | hypothetical protein FLJ21313 |
| FLJ32104 | hypothetical protein FLJ32104 |
| FLJ90586 | hypothetical protein FLJ90586 |
| HRB2 | HIV-1 rev binding protein 2 |
| HSPC022 | HSPC022 protein |
| KIAA0186 | KIAA0186 gene product |
| KIAA0864 | KIAA0864 protein |
| KIAA0937 | KIAA0937 protein |
| KIAA1039 | KIAA1039 protein |
| KIAA1102 | KIAA1102 protein |
| KIAA1337 | KIAA1337 protein |
| KIAA1474 | KIAA1474 protein |
| KIAA1500 | KIAA1500 protein |
| KIAA1695 | hypothetical protein FLJ22297 |
| KIAA1946 | KIAA1946 protein |
| LCN7 | Lipocalin 7 |
| LOC132671 | LOC132671 |
| LOC51219 | clone FLB5214 |
| LOC51659 | HSPC037 protein |
| LOC92689 | hypothetical protein BC001096 |
| MGC10796 | hypothetical protein MGC10796 |
| MSTP031 | MSTP031 protein |
| NDRG3 | NDRG family member 3 |
| NPD009 | NPD009 protein |
| PRO1331 | hypothetical protein PRO1331 |
| REN | likely ortholog of mouse induced by retinoic acid, EGF and NGF |
| TP53INP1 | tumor protein p53 inducible nuclear protein 1 |
|  |  |
|  |  |
| Genes overexpressed in p53^-/-^ HMEC lines over p53^+/+^ HMEC | |
|  |  |
| Cytoskeleton |  |
| MAP2 | microtubule-associated protein 2 |
| MAP7 | microtubule-associated protein 7 |
| Spir-1 | Spir-1 protein; Actin nucleation protein |
| TUBE | epsilon-tubulin |
|  |  |
| ECM and Cell-Cell Communication | |
| ANPEP | alanyl (membrane) aminopeptidase (aminopeptidase N, aminopeptidase M, microsomal aminopeptidase, CD13, p150) |
| APBB2 | amyloid beta (A4) precursor protein-binding, family B, member 2 (Fe65-like) |
| APOE | apolipoprotein E |
| BACE2 | beta-site APP-cleaving enzyme 2 |
| COL1A1 | collagen, type I, alpha 1 |
| COL5A2 | collagen, type V, alpha 2 |
| CRELD1 | cysteine-rich with EGF-like domains 1 |
| DSC2 | desmocollin 2 |
| E48 | lymphocyte antigen 6 complex, locus D |
| F2R | coagulation factor II (thrombin) receptor |
| HLA-B | major histocompatibility complex, class I, B |
| HLA-C | major histocompatibility complex, class I, C |
| HLA-F | major histocompatibility complex, class I, F |
| HLA-G | HLA-G histocompatibility antigen, class I, G |
| K6HF | cytokeratin type II |
| KRT16 | keratin 16 (focal non-epidermolytic palmoplantar keratoderma) |
| MFAP2 | microfibrillar-associated protein 2 |
| MGC4809 | serologically defined breast cancer antigen NY-BR-20 |
| NAV2 | neuron navigator 2 |
| P4HA2 | procollagen-proline, 2-oxoglutarate 4-dioxygenase (proline 4-hydroxylase), alpha polypeptide II |
| PLOD | procollagen-lysine, 2-oxoglutarate 5-dioxygenase (lysine hydroxylase, Ehlers-Danlos syndrome type VI) |
| PROCR | protein C receptor, endothelial (EPCR) |
| PSK-1 | type I transmembrane receptor (seizure-related protein) |
| RSN | restin (Reed-Steinberg cell-expressed intermediate filament-associated protein) |
| SERPINA3 | serine (or cysteine) proteinase inhibitor, clade A (alpha-1 antiproteinase, antitrypsin), member 3 |
| STC2 | stanniocalcin 2 |
| SORT1 | sortilin 1; g-secretase substrate |
| THBD | thrombomodulin |
| TSLP | thymic stromal lymphopoietin |
| VLDLR | very low density lipoprotein receptor |
|  |  |
| Metabolism and Homeostasis | |
| ALDOC | aldolase C, fructose-bisphosphate |
| ARG2 | arginase, type II |
| ASNS | asparagine synthetase |
| BCAT1 | branched chain aminotransferase 1, cytosolic |
| CBS | cystathionine-beta-synthase |
| CTH | cystathionase (cystathionine gamma-lyase) |
| CYC1 | cytochrome c-1 |
| ENO2 | enolase 2, (gamma, neuronal) |
| FACL1, FACL2 | fatty-acid-Coenzyme A ligase, long-chain 1, fatty-acid-Coenzyme A ligase, long-chain 2 |
| FACL2 | fatty-acid-Coenzyme A ligase, long-chain 2 |
| GFPT1 | glutamine-fructose-6-phosphate transaminase 1 |
| GLDC | glycine dehydrogenase (decarboxylating; glycine decarboxylase, glycine cleavage system protein P) |
| GPX3 | glutathione peroxidase 3 (plasma) |
| HMCS | molybdenum cofactor sulfurase |
| MTHFD2 | methylene tetrahydrofolate dehydrogenase (NAD+ dependent), methenyltetrahydrofolate cyclohydrolase |
| OSBPL1A | oxysterol binding protein-like 1A |
| PRH1 | proline-rich protein HaeIII subfamily 1 |
| PYCR1 | pyrroline-5-carboxylate reductase 1 |
| SLC1A4 | solute carrier family 1 (glutamate/neutral amino acid transporter), member 4 |
| SLC7A8 | solute carrier family 7 (cationic amino acid transporter, y+ system), member 8 |
| SPP2 | sphingosine 1-phosphate phosphohydrolase 2 |
| TXNIP | thioredoxin interacting protein |
| UGCG | UDP-glucose ceramide glucosyltransferase |
|  |  |
| Protein Biogenesis and Turnover | |
| BTG1 | B-cell translocation gene 1, anti-proliferative |
| CTSC | cathepsin C |
| DNAJB6 | DnaJ (Hsp40) homolog, subfamily B, member 6 |
| ENSA | endosulfine alpha |
| FBXO5 | F-box only protein 5 |
| HSPA9B | heat shock 70kDa protein 9B (mortalin-2) |
| LAMP3 | lysosomal-associated membrane protein 3 |
| PSA | phosphoserine aminotransferase |
| SIAH2 | seven in absentia homolog 2 (Drosophila) |
| STCH | stress 70 protein chaperone, microsome-associated, 60kDa |
|  |  |
| Protein Secretion |  |
| ARL4 | ADP-ribosylation factor-like 4 |
|  |  |
| Signal Transduction |  |
| cig5 | vipirin; IFNg-induced GTP binding protein |
| EPHB3 | EphB3 |
| FZD7 | frizzled homolog 7 (Drosophila) |
| IL15RA | interleukin 15 receptor, alpha |
| IL1RN | interleukin 1 receptor antagonist |
| LRP16 | LRP16 protein |
| MYLK | myosin, light polypeptide kinase |
| NET1 | neuroepithelial cell transforming gene 1; RHO GTP exchange factor |
| NOTCH3 | Notch homolog 3 (Drosophila) |
| PAWR | PRKC, apoptosis, WT1, regulator |
| STK3 | serine/threonine kinase 3 (STE20 homolog, yeast) |
|  |  |
| Transcription and Translation | |
| AARS | alanyl-tRNA synthetase |
| CBX4 | chromobox homolog 4 (Pc class homolog, Drosophila) |
| CEBPG | CCAAT/enhancer binding protein (C/EBP), gamma |
| DDX18 | DEAD/H (Asp-Glu-Ala-Asp/His) box polypeptide 18 (Myc-regulated) |
| DKC1 | dyskeratosis congenita 1, dyskerin; binds TERC |
| GAS6, SC65 | growth arrest-specific 6, nucleolar autoantigen (55kD) similar to rat synaptonemal complex protein |
| H4F2 | H4 histone, family 2 |
| HDAC3 | histone deacetylase 3 |
| HEY1 | hairy/enhancer-of-split related with YRPW motif 1 |
| HSF1 | heat shock transcription factor 1 |
| IFRD1 | interferon-related developmental regulator 1; binds SIN3 complex |
| JDP2 | jun dimerization protein 2 |
| KLF4 | Kruppel-like factor 4 (gut) |
| LARS | leucyl-tRNA synthetase |
| LGN | LGN protein, binds GPCRs |
| LPHH1 | latrophilin 1; GPCR |
| MARS | methionine-tRNA synthetase |
| NEUGRIN | mesenchymal stem cell protein DSC92; neuronal differentiation nuclear factor |
| NFIL3 | nuclear factor, interleukin 3 regulated |
| NOLC1 | nucleolar and coiled-body phosphoprotein 1 |
| NUP155 | nucleoporin 155kDa |
| RPL17 | ribosomal protein L17 |
| SSRP1 | structure specific recognition protein 1 |
| SUPV3L1 | suppressor of var1, 3-like 1 (S. cerevisiae) |
| TCOF1 | Treacher Collins-Franceschetti syndrome 1 |
| TRAP25 | TRAP/Mediator complex component |
| XPOT | exportin, tRNA (nuclear export receptor for tRNAs) |
|  |  |
| Cell Cycle and DNA Replication | |
| BOP1 | block of proliferation 1 |
| CDCA1 | cell division cycle associated 1 |
| CDKN2A | cyclin-dependent kinase inhibitor 2A (melanoma, p16, inhibits CDK4) |
| CDKN2B | cyclin-dependent kinase inhibitor 2B (p15, inhibits CDK4) |
| CDKN3 | cyclin-dependent kinase inhibitor 3 (CDK2-associated dual specificity phosphatase) |
| CENPA | centromere protein A, 17kDa |
| TACC2 | transforming, acidic coiled-coil containing protein 2 |
|  |  |
| Other |  |
| AUTS2 | autism susceptibility candidate 2 |
| C1orf24 | chromosome 1 open reading frame 24 |
| C20orf97 | chromosome 20 open reading frame 97 |
| DKFZP566B183 | DKFZP566B183 protein |
| EBAG9 | estrogen receptor binding site associated, antigen, 9 |
| FEM1B | fem-1 homolog b (C. elegans) |
| FLJ10134 | hypothetical protein FLJ10134 |
| FLJ11196 | acheron |
| FLJ12895 | hypothetical protein FLJ12895 |
| FLJ14007 | hypothetical protein FLJ14007 |
| FLJ20035 | hypothetical protein FLJ20035 |
| FLJ20150 | hypothetical protein FLJ20150 |
| FLJ20360 | hypothetical protein FLJ20360 |
| FLJ20591 | exosome component Rrp41 |
| FLJ20748 | hypothetical protein FLJ20748 |
| FLJ20989 | hypothetical protein FLJ20989 |
| IFIT2 | interferon-induced protein with tetratricopeptide repeats 2 |
| KIAA0703 | KIAA0703 gene product |
| KIAA0830 | KIAA0830 protein |
| KIAA1357 | KIAA1357 protein |
| KIAA1373 | KIAA1373 protein |
| LOC55862 | uncharacterized hypothalamus protein HCDASE |
| LOC56965 | hypothetical protein from EUROIMAGE 1977056 |
| MGC10946 | hypothetical protein MGC10946 |
| MGC12335 | hypothetical protein MGC12335 |
| MGC14801 | hypothetical protein MGC14801 |
| MGC34923 | hypothetical protein MGC34923 |
| MGC4504 | hypothetical protein MGC4504 |
| PTD015 | PTD015 protein |
