# Supplementary material for: Transcriptional changes associated with breast cancer occur as normal human mammary epithelial cells overcome senescence barriers and become immortalized
Source: Mol Cancer. 2007 Jan 18;6:7. doi: 10.1186/1476-4598-6-7 (PMC1784108; doi:10.1186/1476-4598-6-7)
Supplement: Additional file 4 — Table s4. Gene Expression changes of p53+ cell lines 184A1 versus 184B5. Compilations of genelists and expression statistics of genes expressed uniquely in two p53 wild type HMEC cell lines. [file 1476-4598-6-7-S4.doc]

| **Table s4. Gene Expression changes of p53^+^ cell lines 184A1 versus 184B5** | | |
| --- | --- | --- |
|  |  |  |
| Gene Family and Name | Description | Ratio* |
|  |  |  |
| (1) Signal transduction |  |  |
|  |  |  |
| Ligands and secreted factors | |  |
| IGFBP7 | insulin-like growth factor binding protein 7 | 52 |
| EREG | Epiregulin, EGF-like |  |
| DNER | delta-notch-like EGF repeat-containing transmembrane | 8 |
| NMU | GPR66 ligand, obesity | 6 |
|  |  |  |
| IGFBP4 | Insulin-like binding protein 4 | 0.06 |
| CXCL14 | chemokine (C-X-C motif) ligand 14 | 0.07 |
| EDIL3 | EGF-like repeats and discoidin I-like domains 3 | 0.12 |
| SFRP1 | secreted frizzled-related protein 1 | 0.12 |
| CXCL1, CXCL2 | chemokine (C-X-C motif) ligand 1 (melanoma growth stimulating activity, alpha), chemokine (C-X-C motif) ligand 2 | 0.15 |
| IL8 | interleukin 8 | 0.18 |
| CXCL1 | chemokine (C-X-C motif) ligand 1 (melanoma growth stimulating activity, alpha) | 0.21 |
| DTR | diphtheria toxin receptor (heparin-binding epidermal growth factor-like growth factor) | 0.25 |
|  |  |  |
| Receptors and signaling proteins | |  |
| HRASLS3 | HRAS-like suppressor 3 | 29 |
| IRS1 | insulin receptor substrate 1 | 25 |
| PKIB | protein kinase (cAMP-dependent, catalytic) inhibitor beta | 9 |
| TNFAIP2 | tumor necrosis factor, alpha-induced protein 2 | 8 |
|  |  |  |
| DUSP1 | dual specificity phosphatase 1 | 0.08 |
| GPR | putative G protein coupled receptor | 0.22 |
| IFNGR1 | interferon gamma receptor 1 | 0.24 |
|  |  |  |
| (2) Transcription and translation | |  |
| FABP4 | fatty acid binding protein 4, adipocyte | 47 |
| KOC1 | IGF-II mRNA-binding protein 3 | 9 |
| H2BFS,H2BFT | H2B histone family, member S, H2B histone family, member T | 6 |
| H1F2 | H1 histone family, member 2 | 6 |
| FKSG14 | leucine zipper protein FKSG14 | 6 |
| H2AFO | H2A histone family, member O, H2A histone family, member Q | 5 |
| H1F0 | H1 histone family, member 0 | 4 |
| H2BFT | H2B histone family, member T | 4 |
|  |  |  |
| ATF3 | activating transcription factor 3 | 0.21 |
|  |  |  |
| (2) ECM |  |  |
| Proteases |  |  |
| MMP2 | matrix metalloproteinase 2 (gelatinase A, 72kDa gelatinase, 72kDa type IV collagenase) | 10 |
|  |  |  |
| KLK5 | Kallikrein 5 | 0.04 |
| SERPINB2 | Serine (or cysteine) proteinase inhibitor, clade B (ovalbumin), member2 | 0.06 |
| CTSB | cathepsin B | 0.09 |
| KLK8 | kallikrein 8 (neuropsin/ovasin) | 0.1 |
| SERPINB13 | serine (or cysteine) proteinase inhibitor, clade B (ovalbumin), member 13 | 0.14 |
| SERPINB3 | serine (or cysteine) proteinase inhibitor, clade B (ovalbumin), member 3 | 0.17 |
| CTSC | cathepsin C | 0.21 |
| SERPINB4 | serine (or cysteine) proteinase inhibitor, clade B (ovalbumin), member 4 | 0.25 |
| SERPINB7 | serine (or cysteine) proteinase inhibitor, clade B (ovalbumin), member 7 | 0.25 |
| Structural and secreted proteins | |  |
| AGR2 | anterior gradient 2 homolog (Xenepus laevis) | 20 |
| FBLN1 | fibulin 1, Integrin and nidogen binding protein | 17 |
| LCP1 | lymphocyte cytosolic protein 1 (L-plastin) | 10 |
| FBN1 | fibrillin 1 (Marfan syndrome) | 8 |
| EPS8 | Epidermal growth factor receptor pathway substrate 8 | 8 |
| SPARC | secreted protein, acidic, cysteine-rich (osteonectin) | 7 |
| TNC | tenascin C (hexabrachion) | 6 |
| PCSK1N | Proprotein convertase subtilisin/kexin type 1 inhibitor | 5 |
| MGP` | Matrix Gla protein | 4 |
|  |  |  |
| KRTHB1 | Keratin, hair, basic, 1 | 0.02 |
| SPP1 | Serected phosphoprotein1 (osteopontin, bone sialoprotein, I, early T-lymphocyte activation 1 | 0.04 |
| PI3 | Protease inhibitor 3, (SKALP) | 0.06 |
| CSPG2 | chondroitin sulfate proteoglycan 2 (versican) | 0.07 |
| FN1 | Fibronectin 1 | 0.07 |
| MAGP2 | Microfibril-associated glycoprotein-2 | 0.12 |
| KRT14 | keratin 14 (epidermolysis bullosa simplex, Dowling-Meara, Koebner) | 0.16 |
| THBD | thrombomodulin | 0.21 |
|  |  |  |
| (4) Cytoskeleton |  |  |
| FN1 | fibronectin 1 | 0.07 |
| CALML3 | calmodulin-like 3 | 0.07 |
| TAGLN | transgelin | 0.15 |
| MAIL | molecule possessing ankyrin repeats induced by lipopolysaccharide (MAIL), homolog of mouse | 0.15 |
|  |  |  |
| (5) Interferon responsive |  |  |
| IFI27 | interferon, alpha-inducible protein 27 | 52 |
| G1P3 | interferon, alpha-inducible protein (clone IFI-6-16) | 5 |
|  |  |  |
| (6) Apoptosis |  |  |
| ASC/PYCARD | apoptosis-associated speck-like protein containing a CARD | 10 |
| *Ratio is the fold change of gene expression changes of 184A1 over 184B5. | |  |
