# Supplementary material for: Transcriptional changes associated with breast cancer occur as normal human mammary epithelial cells overcome senescence barriers and become immortalized
Source: Mol Cancer. 2007 Jan 18;6:7. doi: 10.1186/1476-4598-6-7 (PMC1784108; doi:10.1186/1476-4598-6-7)
Supplement: Additional file 5 — Table s5. Gene Expression changes of p53+ cell lines 184A1 versus 184B5. Compilations of genelists and expression statistics of genes expressed uniquely in two p53- HMEC cell lines. [file 1476-4598-6-7-S5.doc]

| **Table s5. Gene Expression changes of p53^+^ cell lines 184A1 versus 184B5** | | |
| --- | --- | --- |
| Gene Family | Description | Ratio* |
|  |  |  |
| (1) Transcription and Translation | |  |
| MLAT4 | myxoid liposarcoma associated protein 4 | 4.63 |
|  |  |  |
| SSA2 | Sjogren syndrome antigen A2 (60kDa, ribonucleoprotein autoantigen SS-A/Ro) | 0.22 |
| ANGPTL4 | angiopoietin-like 4 | 0.22 |
| LRRFIP1 | leucine rich repeat (in FLII) interacting protein 1 | 0.24 |
| SDC3 | syndecan 3 (N-syndecan) | 0.24 |
| DREV1 | CGI-81 protein | 0.24 |
| ALEX2 | armadillo repeat protein ALEX2 | 0.25 |
|  |  |  |
| (2) Signal Transduction | |  |
|  |  |  |
| Ligands and secreted factors | |  |
| DKK1 | dickkopf homolog 1 (Xenopus laevis) | 25.75 |
| IGFBP3 | insulin-like growth factor binding protein 3 | 20 |
| CX3CL1 | chemokine (C-X3-C motif) ligand 1 | 11.67 |
| IL1F9 | interleukin 1 family, member 9 | 9.71 |
| S100A7 | S100 calcium binding protein A7 (psoriasin 1) | 9.25 |
| CXCL16 | chemokine (C-X-C motif) ligand 16 | 8.71 |
| LTB | lymphotoxin beta (TNF superfamily, member 3) | 8.14 |
| IGFBP4 | insulin-like growth factor binding protein 4 | 7.89 |
| IL1B | interleukin 1, beta | 7.86 |
| NRG1 | neuregulin 1, ERB-B2/HER2neu ligand | 7.33 |
| PDGFA | platelet-derived growth factor alpha polypeptide | 6.33 |
| CYR61 | cysteine-rich, angiogenic inducer, 61 | 6.1 |
| HBP17 | heparin-binding growth factor binding protein | 5.64 |
| GAS6, SC65 | growth arrest-specific 6, nucleolar autoantigen (55kD) similar to rat synaptonemal complex protein | 5.5 |
| JAG1 | jagged 1 (Alagille syndrome) | 5.18 |
| FGF11 | fibroblast growth factor 11 | 4.88 |
| VEGFC | vascular endothelial growth factor C | 4.52 |
| IGFBP2 | insulin-like growth factor binding protein 2, 36kDa | 4.46 |
| CKLFSF7 | chemokine-like factor super family 7 | 4.1 |
| BMP1 | bone morphogenetic protein 1 | 4 |
|  |  |  |
| CCL20 | chemokine (C-C motif) ligand 20 | 0.04 |
| TFPI2 | tissue factor pathway inhibitor 2 | 0.06 |
| SFRP1 | secreted frizzled-related protein 1 | 0.08 |
| IL18 | interleukin 18 (interferon-gamma-inducing factor) | 0.11 |
| S100P | S100 calcium binding protein P | 0.17 |
| S100A9 | S100 calcium binding protein A9 (calgranulin B) | 0.19 |
| WNT5A | wingless-type MMTV integration site family, member 5A | 0.19 |
| LTBP1 | latent transforming growth factor beta binding protein 1 | 0.21 |
|  |  |  |
| Receptors and Signaling Components | |  |
| TNFRSF21 | tumor necrosis factor receptor superfamily, member 21 | 18.25 |
| FEZ1 | fasciculation and elongation protein zeta 1 (zygin I) | 13.33 |
| PARG1 | PTPL1-associated RhoGAP 1 | 11.5 |
| FGFR2 | fibroblast growth factor receptor 2 (bacteria-expressed kinase, keratinocyte growth factor receptor, craniofacial dysostosis 1, Crouzon syndrome, Pfeiffer syndrome, Jackson-Weiss syndrome) | 9 |
| TNFRSF21 | tumor necrosis factor receptor superfamily, member 21 | 8.4 |
| ARTN | artemin | 7.75 |
| TNFSF10 | tumor necrosis factor (ligand) superfamily, member 10 | 7.69 |
| PTPRZ1 | protein tyrosine phosphatase, receptor-type, Z polypeptide 1 | 7.67 |
| AXL | AXL receptor tyrosine kinase | 7.52 |
| PPP1R3C | protein phosphatase 1, regulatory (inhibitor) subunit 3C | 7.38 |
| CCND2 | cyclin D2 | 6.33 |
| G0S2 | putative lymphocyte G0/G1 switch gene | 6.17 |
| NOTCH1 | Notch homolog 1, translocation-associated (Drosophila) | 5.67 |
| DDR1 | discoidin domain receptor family, member 1 | 5.28 |
| RDC1 | G protein-coupled receptor | 5.14 |
| PTPRF | protein tyrosine phosphatase, receptor type, F | 5.13 |
| PPP1R14C | protein phosphatase 1, regulatory (inhibitor) subunit 14C | 4.85 |
| TIP-1 | Tax interaction protein 1 | 4.29 |
| CDC42EP3 | CDC42 effector protein (Rho GTPase binding) 3 | 4.16 |
| CHRNB1 | cholinergic receptor, nicotinic, beta polypeptide 1 (muscle) | 4 |
| SNK | serum-inducible kinase | 4 |
|  |  |  |
| SAMSN1 | SAM domain, SH3 domain and nuclear localisation signals, 1 | 0.03 |
| FKBP5 | FK506 binding protein 5 | 0.04 |
| DUSP1 | dual specificity phosphatase 1 | 0.05 |
| BIRC3 | baculoviral IAP repeat-containing 3 | 0.05 |
| TOP1 | topoisomerase (DNA) I | 0.06 |
| NUCKS | similar to rat nuclear ubiquitous casein kinase 2 | 0.09 |
| NFKBIA | nuclear factor of kappa light polypeptide gene enhancer in B-cells inhibitor, alpha | 0.09 |
| PK428 | Ser-Thr protein kinase related to the myotonic dystrophy protein kinase | 0.1 |
| TRAP150 | thyroid hormone receptor-associated protein, 150 kDa subunit | 0.11 |
| TREM1 | triggering receptor expressed on myeloid cells 1 | 0.11 |
| *Ratio is the fold change of gene expression changes of 184AA2 over 184AA3. | |  |
