# Supplementary material for: Transcriptional changes associated with breast cancer occur as normal human mammary epithelial cells overcome senescence barriers and become immortalized
Source: Mol Cancer. 2007 Jan 18;6:7. doi: 10.1186/1476-4598-6-7 (PMC1784108; doi:10.1186/1476-4598-6-7)
Supplement: Additional file 6 — Table s6. Gene Expression Changes Resulting from Expression of ERB-B2/Her2neu in 184B5. Compilations of genelists and expression statistics of genes that change expression following overexpression of the Her-2neu oncogene. [file 1476-4598-6-7-S6.doc]

| **Table s6. Gene Expression Changes Resulting from Expression of ERB-B2/Her2^neu^ in 184B5** | | |
| --- | --- | --- |
|  |  |  |
| Gene Family | Description | Ratio* |
|  |  |  |
| (1) Signal Transduction | |  |
|  |  |  |
| Ligands and secreted factors | |  |
| IL24 | interleukin 24 | 22.11 |
| TFPI2 | tissue factor pathway inhibitor 2 | 4.27 |
|  |  |  |
| IGFBP4 | insulin-like growth factor binding protein 4 | 0.05 |
| CXCL14 | chemokine (C-X-C motif) ligand 14 | 0.05 |
| CXCL1, CXCL2 | chemokine (C-X-C motif) ligand 1 (melanoma growth stimulating activity, alpha), chemokine (C-X-C motif) ligand 2 | 0.08 |
| TNFSF10 | tumor necrosis factor (ligand) superfamily, member 10 | 0.11 |
| DLL1 | delta-like 1 (Drosophila) | 0.13 |
| TGFB2 | transforming growth factor, beta 2 | 0.16 |
| DKK3, RIG | dickkopf homolog 3 (Xenopus laevis), regulated in glioma | 0.2 |
| CCL20 | chemokine (C-C motif) ligand 20 | 0.22 |
| CXCL14 | chemokine (C-X-C motif) ligand 14 | 0.22 |
| S100A8 | S100 calcium binding protein A8 (calgranulin A) | 0.24 |
|  |  |  |
| Receptors and other membrane proteins | |  |
| SPRR1A | small proline-rich protein 1A | 0.23 |
| SH3BGRL | SH3 domain binding glutamic acid-rich protein like | 0.24 |
| RDC1 | G protein-coupled receptor | 0.24 |
| MLP | MARCKS-like protein | 0.24 |
| DRAPC1 | hypothetical protein DRAPC1 | 0.25 |
|  |  |  |
| AKAP12 | A kinase (PRKA) anchor protein (gravin) 12 | 10.88 |
| DNAJB6 | DnaJ (Hsp40) homolog, subfamily B, member 6 | 4.13 |
| PPFIA1 | protein tyrosine phosphatase, receptor type, f polypeptide (PTPRF), interacting protein (liprin), alpha 1 | 4 |
| HRASLS3 |  | 4 |
|  |  |  |
| MYL9 | myosin, light polypeptide 9, regulatory | 0.08 |
| TREM2 | triggering receptor expressed on myeloid cells 2 | 0.13 |
| FEZ1 | fasciculation and elongation protein zeta 1 (zygin I) | 0.16 |
| EFNA1 | ephrin-A1 | 0.16 |
| DUSP1 | dual specificity phosphatase 1 | 0.16 |
|  |  |  |
|  |  |  |
| (2) Transcription and gene expression | |  |
| FOS | v-fos FBJ murine osteosarcoma viral oncogene homolog | 0.21 |
| ZFP36 | zinc finger protein 36, C3H type, homolog (mouse) | 0.24 |
|  |  |  |
| (3) ECM and Cell-Cell Communication | |  |
|  |  |  |
| Proteases |  |  |
| CST6 | cystatin E/M | 12.5 |
| PLAT | plasminogen activator, tissue | 6.29 |
| KLK6 | kallikrein 6 (neurosin, zyme) | 5.27 |
|  |  |  |
| SERPINB3 | serine (or cysteine) proteinase inhibitor, clade B (ovalbumin), member 3 | 0.2 |
| SERPINB4 | serine (or cysteine) proteinase inhibitor, clade B (ovalbumin), member 4 | 0.21 |
| SPUVE | protease, serine, 23 | 0.23 |
|  |  |  |
| Structural and Secreted Proteins | |  |
| ESDN | endothelial and smooth muscle cell-derived neuropilin-like protein | 8.5 |
| COL13A1 | collagen, type XIII, alpha 1 | 6.22 |
| SCEL | sciellin | 4.67 |
| PLAC8 | placenta-specific 8 | 4.63 |
| KRT8 | keratin 8 | 4.56 |
|  |  |  |
| SPRR1B | small proline-rich protein 1B (cornifin) | 0.12 |
| GJB2 | gap junction protein, beta 2, 26kDa (connexin 26) | 0.12 |
| KRT6B | keratin 6B | 0.12 |
| SEMA3C | sema domain, immunoglobulin domain (Ig), short basic domain, secreted, (semaphorin) 3C | 0.15 |
| C1R | complement component 1, r subcomponent | 0.16 |
| FLRT3 | fibronectin leucine rich transmembrane protein 3 | 0.16 |
| KRT16 | keratin 16 (focal non-epidermolytic palmoplantar keratoderma) | 0.17 |
| FLRT3 | fibronectin leucine rich transmembrane protein 3 | 0.21 |
| ITGB6 | integrin, beta 6 | 0.21 |
| PI3 | protease inhibitor 3, skin-derived (SKALP) | 0.22 |
| T1A-2 | lung type-I cell membrane-associated glycoprotein | 0.22 |
| DSC3 | desmocollin 3 | 0.23 |
| CSPG2 | chondroitin sulfate proteoglycan 2 (versican) | 0.23 |
| PCDH19 | protocadherin 19 | 0.25 |
|  |  |  |
| (5) Cytoskeleton |  |  |
| HPCAL1 | hippocalcin-like 1 | 4.65 |
|  |  |  |
| TAGLN | transgelin | 0.07 |
| BPAG1 | bullous pemphigoid antigen 1, 230/240kDa | 0.18 |
| DD96 | epithelial protein up-regulated in carcinoma, membrane associated protein 17 | 0.2 |
| NS1-BP | NS1-binding protein | 0.25 |
|  |  |  |
| (6) Metabolism |  |  |
| ASNS | asparagine synthetase | 7.89 |
|  |  |  |
| ALDH3A1 | aldehyde dehydrogenase 3 family, memberA1 | 0.19 |
| CA12 | carbonic anhydrase XII | 0.22 |
| AKR1C3 | aldo-keto reductase family 1, member C3 (3-alpha hydroxysteroid dehydrogenase, type II) | 0.22 |
|  |  |  |
| (7) IFN-Regulated Genes | |  |
| IFIT1 | interferon-induced protein with tetratricopeptide repeats 1 | 0.06 |
|  |  |  |
| *Ratio is the fold change of gene expression changes of 184B5ME over 184B5. | | |
